# Inactivation of Pseudovirus Expressing the D614G Spike Protein Mutation using Nitric Oxide‐Plasma Activated Water

**DOI:** 10.1002/advs.202411515

**Published:** 2024-11-13

**Authors:** Paritosh Patel, Neha Kaushik, Tirtha Raj Acharya, Sudakshya S. Lenka, Soujanya Ghosh, Rizwan Wahab, Suresh K. Verma, Eun Ha Choi, Nagendra Kumar Kaushik

**Affiliations:** ^1^ Department of Electrical and Biological Physics Plasma Bioscience Research Center Kwangwoon University Seoul 01897 South Korea; ^2^ Department of Biotechnology College of Engineering The University of Suwon Hwaseong 18323 South Korea; ^3^ School of Biotechnology KIIT University Bhubaneswar Odisha 751024 India; ^4^ Department of Zoology College of Science King Saud University Riyadh 11451 Saudi Arabia

**Keywords:** mutated virus inactivation, nitric oxides, plasma activated water, SARS‐CoV‐2 pseudovirus D614G spike mutant, variants of concern

## Abstract

Variants of concern (VOCs) of Severe Acute Respiratory Syndrome Coronavirus 2 (SARS‐CoV‐2) exhibit high infectivity due to mutations, particularly in the spike protein, that facilitate enhanced binding of virus to human angiotensin‐converting enzyme 2 (hACE2). The D614G mutation, situated in S1‐domain, promotes the open conformation of spike protein, augmenting its interaction with hACE2. Activated water neutralizes pathogens by damaging biological molecules; however, its effect on mutated SARS‐CoV‐2 or VOCs requires further exploration. Here, the efficacy of nitric oxide (NO_x_)‐plasma activated water (PAW) in inhibiting infections by SARS‐CoV‐2 pseudovirus expressing D614G‐mutated spike protein is investigated, which serves as a model for mutated SARS‐CoV‐2. Results demonstrated high prevalence of D614G mutation in SARS‐CoV‐2 and its VOCs. NO_x_‐PAW is non‐toxic to cells at high concentration, inhibiting infection by 71%. Moreover, NO_x_‐PAW induced structural changes in S1‐domain of spike protein, reducing its binding affinity and lowering clathrin‐mediated endocytosis‐related gene expression. Additionally, in silico analysis revealed NO_x_ species in NO_x_‐PAW played key role in impairing S1‐domain function of the mutated SARS‐CoV‐2 pseudovirus by interacting directly with it. Collectively, these findings reveal the potent inactivation ability of PAW against mutated SARS‐CoV‐2 and suggest its potential application in combating emerging variants of SARS‐CoV‐2 and other viral threats.

## Graphical abstract

SARS‐CoV‐2 mutants are of concern. The inactivation of mutated viruses by plasma‐activated water remains unclear. In this study, NO_x_‐PAW demonstrates inactivation of SARS‐CoV‐2 pseudovirus with D614G mutation by interacting with the amino acids in S1‐domain of mutated spike protein. Furthermore, RNS destabilizes S1‐domain leading to loss of bio‐functionality. This hinders the entry of viruses in host cells through CME pathway.

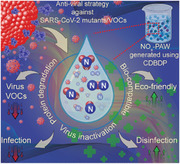

## 1 Introduction

In the last century, three coronaviruses have crossed the species barrier: Middle East respiratory syndrome coronavirus, severe acute respiratory syndrome coronavirus (SARS‐CoV), and SARS‐CoV‐2. SARS‐CoV‐2 caused the coronavirus disease (COVID‐19) pandemic, which originated in Wuhan, China in 2019 and has resulted in 704 million cases and 7 million deaths as of February 2024.^[^ ^1^ ^]^ The development of drugs against SARS‐CoV‐2 and its variants is still underway. These drugs target crucial viral or host proteins for viral replication^[^ ^2^, ^3^, ^4^, ^5^ ^]^ and utilize either the original virions or engineered mRNA‐encoding viral proteins to elicit an immune response against viral infections.^[^ ^6^, ^7^, ^8^ ^]^ However, the high mutation rate of viruses diminishes the efficacy of drugs against specific viral strains, thereby preventing complete protection. Strategies for managing viral spread are vital for preventing direct transmission. Moreover, addressing indirect transmission risks is essential for effective comprehensive infection control.

The predominant modes of transmission for SARS‐CoV‐2 involve the inhalation of droplets and aerosols (direct transmission) or large droplets (fomites) contaminating surfaces (indirect transmission).^[^ ^9^, ^10^ ^]^ Furthermore, SARS‐CoV‐2 can persist on surfaces, which supports surface‐mediated transmission. Human coronavirus 229E found on diverse surfaces, including stainless steel, glass, and ceramics, retains infectivity.^[^ ^11^ ^]^ The delta variant (SARS‐CoV‐2) exhibits heightened transmissibility than the alpha variant, possibly because of its higher persistence.^[^ ^12^ ^]^ Porous and nonporous fomites facilitate viral dissemination. Factors such as porosity, temperature, adsorption, evaporation, isoelectric point, and hydrophobicity of the contaminated surface influence viral spread dynamics. Understanding these mechanisms is crucial for developing effective infection control measures to mitigate the risk of indirect transmission and prevent the spread of SARS‐CoV‐2 in diverse environments and settings.

Environmental factors and natural selection have resulted in the development of numerous mutations in the SARS‐CoV‐2 genome, resulting in the emergence of several variants of concerns (VOCs). These VOCs have the potential to contaminate surfaces and environments, which augments their persistence, stability, and transmissibility.^[^ ^13^, ^14^, ^15^ ^]^ This may play a critical role in the indirect transmission of SARS‐CoV‐2 via fomites. The D614G mutation, found in the spike region of SARS‐CoV‐2, has increased in prevalence among SARS‐CoV‐2 variants.^[^ ^16^ ^]^ Plante et al. found that the G614 variant exhibited higher infectivity as temperature increased/decreased, implying high stability.^[^ ^17^ ^]^ Additionally, Yang et al. suggested that the G614 mutation enhances spike protein fitness by reducing its sensitivity to temperature‐induced denaturation.^[^ ^18^ ^]^ These findings underscore the significance of the D614G mutation in the transmission and persistence of SARS‐CoV‐2 variants.

Non‐thermal plasma (NTP) treatment is widely recognized for its ability to inactivate various pathogens, including bacteria like *Pseudomonas aeruginosa* and *Streptococcus mutans*,^[^ ^19^, ^20^ ^]^ viruses such as SARS‐CoV‐2 and HCoV‐229E,^[^ ^21^, ^22^, ^23^, ^24^ ^]^ and fungi like *Ascosphaera apis* and dermatophytes micromycetes.^[^ ^25^, ^26^ ^]^ This inactivation is typically achieved by directly exposing these pathogens to NTP. However, to make this technology more practical and applicable in real‐world scenarios, plasma‐activated water (PAW) has been developed. PAW contains reactive oxygen and nitrogen species (RONS). Studies have shown that PAW is effective at inactivating viruses. For example, Michael G. Kong et al. found short‐lived like ONOO^−^ in PAW, resulting in reduction of the relative luminescence units (RLU) of treated Pseudovirus (SARS‐CoV‐2 S protein) by 500 times compared to untreated samples.^[^ ^27^ ^]^ Similarly, Hongbo Qin et al. observed involvement of ONOO^−^, ^1^O_2_, O_2_^−^ and ·OH species in decreasing TCID_50_ to 99.94% after 300 s of NTP exposure.^[^ ^22^ ^]^ Another study reported reduction of T4 bacteriophage from 5.8 × 10^11^ to 6.0 × 10^6^ PFU mL^−1^ after 1 h of PAW exposure.^[^ ^28^ ^]^ Gomez‐Casado et al. demonstrated viral inactivation following 30 min of PAW treatment.^[^ ^29^ ^]^ Although, PAW has been studied for its ability to inactivate viruses like (SARS‐CoV‐2 and H1N1). However, due to the mutations in their amino acids, the emergence of SARS‐CoV‐2 VOCs presents new challenges. Leading to high environmental persistence and requirement of prolonged and excessive disinfectant usage, negatively impacting the environment. Similarly, studies involved prolonged exposure times with PAW to achieve significant viral inactivation.^[^ ^28^, ^29^ ^]^ Second, short‐lived reactive species generated during plasma treatment are primarily responsible for viral inactivation.^[^ ^22^, ^27^ ^]^ These species interact with the amino acids of the virus, leading to inactivation.

Despite these promising results, many of these studies relied on either prolonged exposure time or short‐lived RONS for viral inactivation. The short lifespan of such reactive species poses a limitation (it cannot be stored for a longer time for practical implication), especially in the context of the high persistence rate of SARS‐CoV‐2 VOCs. Therefore, for effective viral inactivation, it is crucial that these reactive species not only have an extended lifespan but also interact with viral components more efficiently. To overcome this challenge, plasma‐generated nitric oxide water (NO_x_‐PAW) has been proposed, which is enriched with relatively stable, long‐lived reactive nitrogen species and has shown efficacy in microbial inactivation.

Recently, our lab developed a cylindrical‐based dielectric barrier discharge plasma (CDBDP) has been employed to generate NO_x_‐PAW for inactivating SARS‐CoV‐2 VOCs, a novel process developed in our laboratory, containing stable, long‐lived reactive nitrogen species such as NO, NO_2_, NO_3,_ and N_2_O.^[^ ^30^ ^]^ While studies suggest its potential for microbial inactivation,^[^ ^30^, ^31^ ^]^ However, its effectiveness against SARS‐CoV‐2 and its VOCs has not yet been thoroughly investigated, especially in molecular mechanical aspects, highlighting the need for NO_x_‐PAW in further research. Alternate disinfection methods exhibited varying degrees of effectiveness against SARS‐CoV‐2 and related viruses. UV‐C irradiation reduces SAR‐CoV‐2 by 1 log10, UV‐B lowers Vaccinia virus by 2 log10 after 34 min to 2 h,^[^ ^32^ ^]^ dry heat achieves 1–5 log10 reduction in 30–120 min,^[^ ^32^ ^]^ ozone reduces SARS‐CoV‐2 by 90% in 30 min and chlorine, peracetic acid and hydrogen peroxide achieved 4.75 log10, 4 log10, and over 4 log10 reduction in 20, 5, and 1 min. Despite their effectiveness, these methods require long exposure or harsh chemicals, making NO_x_‐PAW water a more practical and safer option for real‐world virus inactivation.

This study aimed to explore the inactivation of mutated SARS‐CoV‐2 Pseudovirus using NO_x_‐PAW. This approach served as a model for evaluating the efficacy of NO_x_‐PAW in inactivating mutated strains of SARS‐CoV‐2. This is the first study of its kind to explore the effectiveness of this innovative approach against SARS‐CoV‐2 VOCs, offering new insights into the potential of NO_x_‐PAW for combating emerging viral threats. Furthermore, we revealed the interaction of reactive nitrogen species (RNS) with spike proteins carrying the D614G in S1‐domain, resulting in the protein losing its bio‐functionality, leading to the abrogation of receptor‐mediated endocytosis in host cells.

## 2 Results

### 2.1 Prevalence of the Aspartic Acid (D) to Glycine (G) Mutation at Position 614 (D614G) in SARS‐CoV‐2 Spike Proteins

**Figure 1:**
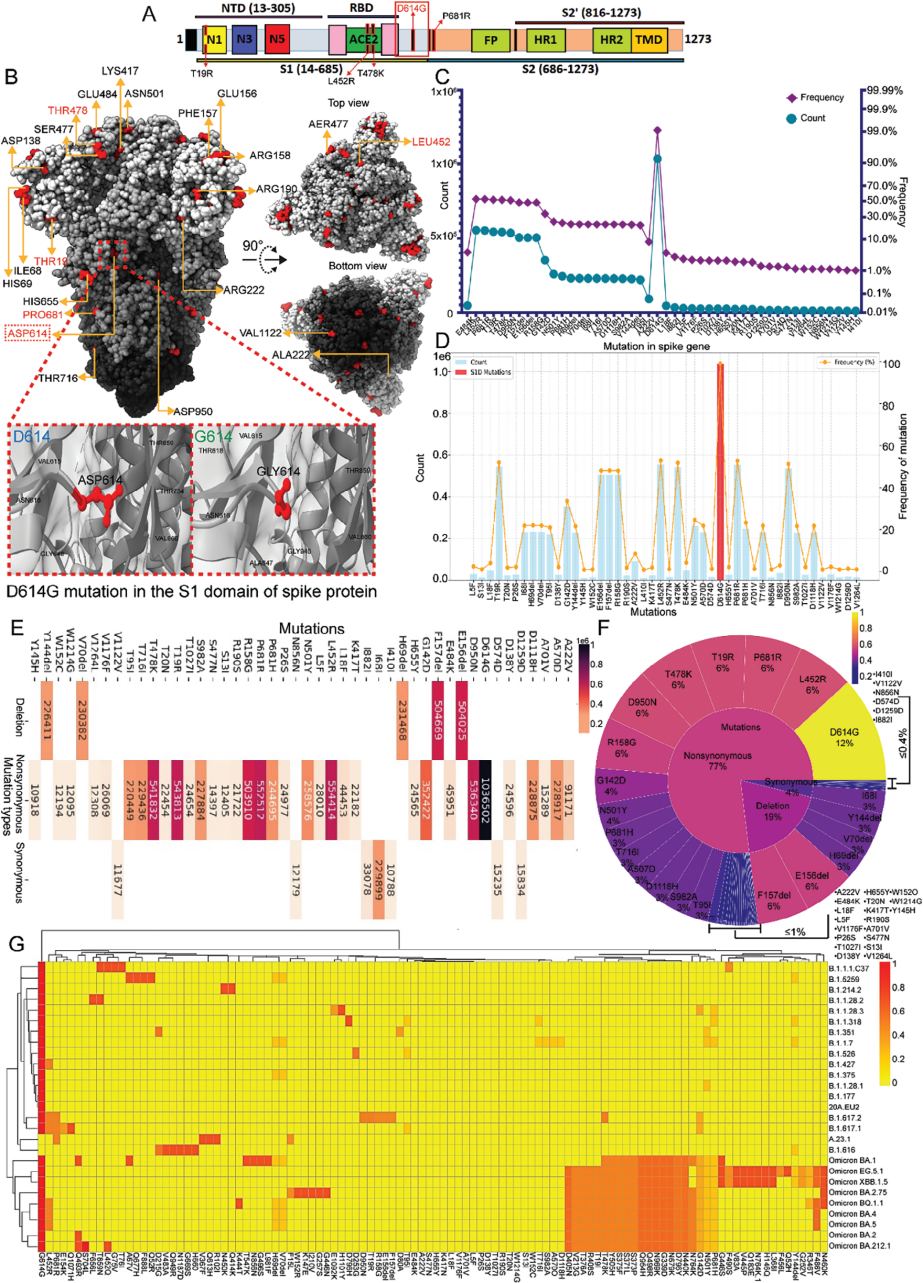
Protein sequence analysis of the Severe Acute Respiratory Syndrome Coronavirus 2 (SARS‐CoV‐2) spike protein. (A) Schematic representation of the domain organization of the spike protein, denoting the most prevalent mutations. (B) The 3D trimeric architecture of the spike protein (PDB ID: 7dzw) variant is shown. Each region is shown at 90° rotations to show the location of the mutations. Graph demonstrating the mutational landscape of the SARS‐CoV‐2 spike protein (C) and S1‐domain (D), based on 1.2 million sequences from the NCBI virus database. Amino acid positions and mutations are shown on the X‐axis, and mutation count and frequency are presented on the Y‐axis. Peaks in the mutation count highlight structurally significant regions. (E) Heat map analysis of mutations across the SARS‐CoV‐2 spike protein. Rows correspond to discrete amino acids within the protein sequence. Columns represent distinct sequences, illustrating variant diversity. The map highlights mutation trends and changes in mutation frequencies in specific regions. Color intensities correlate with mutation frequency, with warmer colors indicating higher mutation rates. (F) Sunburst plot representing mutation types and frequencies. The inner circle is divided into three sectors for different mutation types, while the outer circle is categorized by frequency, corresponding to mutation frequency. Frequency values are shown in the outer sectors, with warmer colors indicating higher occurrence rates. (G) Analysis of SARS‐CoV‐2 spike protein mutations, visualizing genetic variation and enabling identification of mutation hotspots and patterns across viral sequences. Rows correspond to amino acid positions, and columns represent individual variants, with color intensity indicating mutation frequency.

Examination of 1 048 576 nucleotide sequences from SARS‐CoV‐2 spike (S) glycoproteins revealed a total of 46 mutations. Annotation of the complete sequence of the S protein, consisting of 1273 amino acids (aa), delineated the S1‐domain as spanning aa 14‐685, in which five prevalent mutations were identified: aspartic acid 614 to glycine (D614G), leucine 452 to arginine (L452R), proline 681 to arginine (P681R), threonine 19 to arginine (T19R), and threonine 478 to lysine (T478K). The S2 domain is situated within a 686–1273 of the S protein. However, this study focused primarily on the S1‐domain, particularly the five most prevalent mutations (**Figure** **1**A). The SARS‐CoV‐2 S protein (PDB ID: 7dzw) was used as the structural framework for subsequent investigations. The molecular visualization software UCSF ChimeraX facilitated delineation of essential regions, domains, and amino acids (aa) residues within the S protein. Mutations were introduced into the protein structure (PDB ID: 7dzw) using Chimera X. All 46 mutations were highlighted in the molecular structure, along with the five most prevalent mutations (mentioned in red). This revealed their spatial positioning within the protein structure, as illustrated in Figure 1B. A multiparameter plot was generated (Figure 1C) to examine the frequency and types of mutations in the S protein. The left Y‐axis denotes the count, and the right Y‐axis represents the mutation frequency. The graph revealed that, among the 46 mutations observed in 1 048 576 nucleotide sequences from S proteins, the five most prevalent mutations exhibited a frequency of ≥51.8%. Notably, the D614G mutation displayed the highest frequency (99.12%) and was detected in a total of 1 036 502 sequences. Moreover, to assess mutations within the S1‐domain, a dataset comprising 1 048 576 nucleotide sequences (database: NCBI virus) was examined. Figure 1D displays mutations with frequencies exceeding 50% in S1‐domain. Only the D614G mutation met this criterion and is highlighted in the graph by a red bar. Figure 1E visualizes the various types of mutations in the sequences. Most of the mutations were non‐synonymous, involving the replacement of one aa with another, potentially impacting structural integrity and fidelity. In addition to the non‐synonymous mutations, seven synonymous and five deleterious aa mutations were observed. The five most prevalent mutations, D614G, L452R, P681R, T19R, and T478K, were identified as non‐synonymous mutations (Figure S1, Supporting Information). Notably, the prevalence of the D614G mutation is shown in Figure 1F, which illustrates that 12% of all mutations within the sequences correspond to the D614G non‐synonymous mutation. Furthermore, 27 SARS‐CoV‐2 VOCs sourced from the NCBI database were examined to assess the frequency of the D614G mutation (Figure 1G). Among these, the T19R, P681R, L452R, T478K, and D614G mutations were detected in 1, 3, 6, 12, and 25 VOCs, respectively. In summary, the five predominant mutations were located in the S1‐domain, among which the most frequent was the D614G mutation, which exhibited a high frequency among the nucleotide sequences.

### 2.2 Electrical and Optical Characteristics of CDBDP

**Figure 2:**
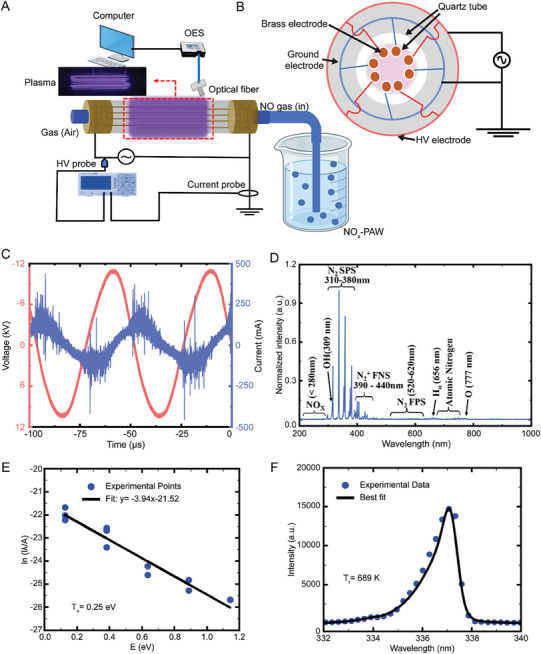
Plasma setup and diagnostic of cylindrical dielectric discharge plasma. (A) Diagram illustrating the configuration of the CDBDP setup, (B) electrode layout, (C) voltage‐current profile, (D) analysis of optical emission spectra (OES), (E) Boltzmann plot analysis for determination of vibrational temperature (T_v_), and (F) optimal fitting of experimental data for calculation of the rotational temperature (T_r_).

CDBDP was employed to generate NO_x_‐PAW water, and the setup is illustrated in **Figure** **2**A,B. Figure 2C depicts the voltage–current characteristics of CDBDP, including the peak voltage (11.1 kV) and peak current (437 mA). With a plasma resonance frequency of 20.9 kHz and 100% duty cycle, a surface temperature of 155 °C was maintained, enabling continuous plasma treatment. The energy dissipation was 0.75 mJ, operating at 15.6 W. Optical emission spectra (OES), shown in Figure 2D, revealed OH radical emission at 309 nm, NO_x_ emission between 200 and 280 nm, and N_2_ SPS (second positive system) emission spanning 311–380 nm. Peaks at 740–750 and 777 nm confirmed the presence of atomic nitrogen (N) and oxygen (O).^[^ ^33^ ^]^ These emissions, which result from water vapor or molecular oxygen dissociation, confirm the presence of atomic O. T_r_ and T_v_ are vital for CDBDP plasma chemistry, particularly for NO production. T_r_ promotes N_2_ dissociation, leading to NO generation via collisional energy transfer. T_v_ enhances N_2_ dissociation, facilitating the formation of N atoms, which react with O_2_ to produce NO.^[^ ^34^ ^]^ Here, T_r_ and T_v_ were measured as 550 K and 0.23 eV (Figure 2E,F), respectively.

### 2.3 Determination of RONS and Physiochemical Characteristics of NO_x_‐PAW

**Figure 3:**
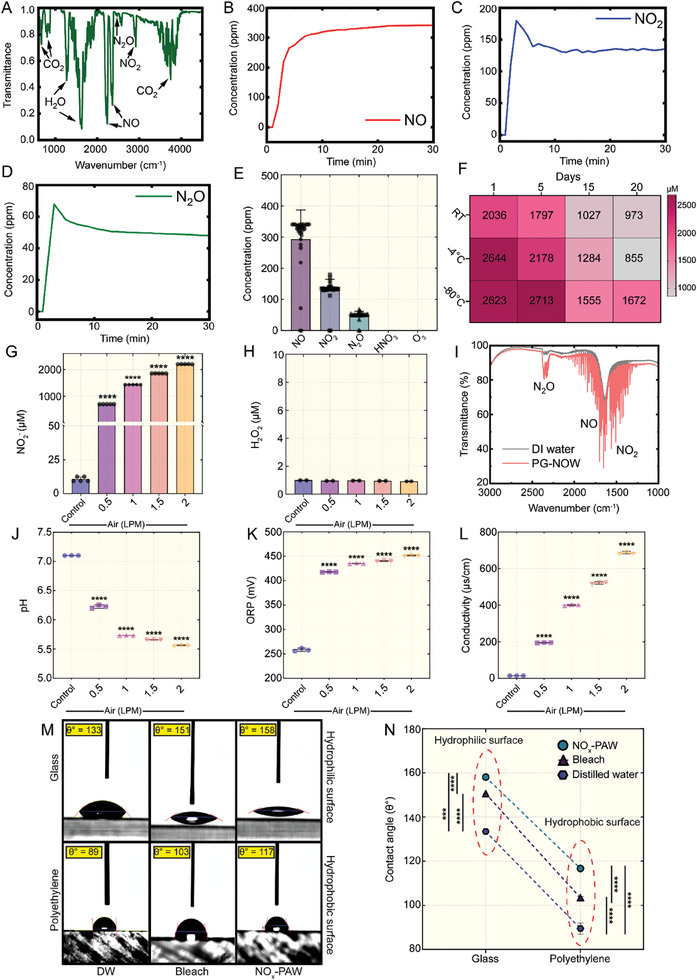
Examination of the physiochemical characteristics and reactive species in cylindrical dielectric barrier discharge plasma. (A) Fourier transform infrared spectroscopy (FTIR) revealed the composition of gases produced in the plasma. Concentrations of (B) NO, (C) NO_2_, and (D) N_2_O determined by FTIR. (E) Time‐averaged concentrations of reactive species over a 30‐min plasma exposure period. (F) Aging effects on plasma‐activated water (PAW). Concentrations of (G) NO_2_^−^ and (H) H_2_O_2_ in PAW. (I) FTIR evaluation of PAW liquid. Evaluation of the (J) pH, (K) oxidation‐reduction potential (ORP), and (L) electrical conductivity of PAW at different times during plasma treatment. (M,N) Contact angle of PAW for distilled water (DW) and bleach on glass and polyethylene surfaces. Statistical analysis between groups was conducted using one‐ and two‐way ANOVA with Dunnett's and Tukey's multiple comparison tests. The level of significance was determined, and stars (^*^) in graphs denote *p*‐values ^(***^ *p* ≤ 0.001 and ^****^ *p* ≤ 0.0001).

Transmission Fourier transform infrared (FTIR) spectroscopy (**Figure** **3**A) was used to analyze gas‐phase RONS in CDBDP. Data were collected every 60 s over 30 min, at wavelengths of 1000–3000 cm^−1^. Between 0 and 2 min, no RONS were detected when the plasma was off. Between 3 and 30 min, NO, NO_2_, and N_2_O were produced, as demonstrated by the distinct spectra: 1700–2000 cm^−1^ (NO), 1540–1660 cm^−1^ and 2840–2940 cm^−1^(NO_2_), and 2160–2260 cm^−1^ (N_2_O) (Figure 3B–D).^[^ ^34^ ^]^ Figure 3E shows the average concentration of reactive species produced by CDBDP during the 30‐min plasma exposure period: 293 ppm NO, 128 ppm NO_2_, and 48 ppm N_2_O, with no HNO_3_ or O_3_ detected.

This study investigated the effects of time on NO_x_‐PAW by monitoring the NO_2_^−^ concentration over 20 d (Figure 3F). Various temperatures (room temperature, 4 and −80 °C) resulted in different NO_2_^−^ levels, with the highest at −80° C. The initial NO_2_^−^ level was 2200 µm with 2 lpm air flow for 30 min of plasma treatment. NO_2_^−^ concentrations fluctuated (706, 1436, 1856, and 2200 µm) with different ambient air flow rates (0.5, 1.0, 1.5, and 2.0 lpm, respectively) in a 30 min CDBDP treatment with 50 mL distilled water (DW) (Figure 3G). Interestingly, the H_2_O_2_ concentration did not significantly change (≈0.92 µm) with changes in the ambient air flow rates (Figure 3H). FTIR analysis revealed decreased transmittance of specific functional groups, indicating chemical alterations induced by plasma treatment and significantly increased NO_x_ species: 1200–1600 cm^−1^ NO, 1600–2100 cm^−1^ NO_2_, and 2250–2450 cm^−1^ N_2_O^[^ ^31^ ^]^ (Figure 3I).

Figure 3J–L illustrates changes in pH, oxidation‐reduction potential (ORP), and electrical conductivity in PAW with varying gas flow rates between 0.5 and 2 lpm (liter per minute). Initially, DW had a pH of 7.10, ORP of 258 mV, and electrical conductivity of 14.6 µS cm^−1^. The pH of PAW decreased as the airflow rate decreased (0.5–2.0 lpm), while the ORP and conductivity increased. After 30 min of CDBDP treatment (2.0 lpm) in 50 mL DW, the pH of PAW dropped to 5.6, the ORP was 452 mV, and conductivity was 687 µS cm^−1^. These alterations result from NO and NO_2_ generation during plasma‐water interactions, leading to the production of acidic compounds and more redox reactions that elevate the ORP^[^ ^35^ ^]^ as well as the formation of ions such as nitrite and nitrate, thereby elevating electrical conductivity.^[^ ^36^ ^]^ Figure 3M,N depict the contact angle variations for DW, bleach, and NO_x_‐PAW on glass and polyethylene surfaces. Initially, DW demonstrated contact angles (θ) of 133° and 89° on glass and polyethylene, respectively. Bleach showed θ of 151° and 103° on glass and polyethylene, while NO_x_‐PAW displayed θ of 158° and 177° on glass and polyethylene, respectively. Plasma generated NO_x_‐PAW, has a reduced contact angle when compared with bleach and DW. This is caused by introduction of functional groups (─OH, C≐O, ─COOH, and ─NH_2_) during plasma treatment, thereby enhancing the surface wettability. This reduced contact angle indicates improved surface characteristics for cleaning.^[^ ^37^ ^]^

### 2.4 Biocompatibility Test of NO_x_‐PAW with HEK‐293T Expressing Human Angiotensin‐Converting Enzyme 2 (HEK‐293T‐hACE2)

**Figure 4:**
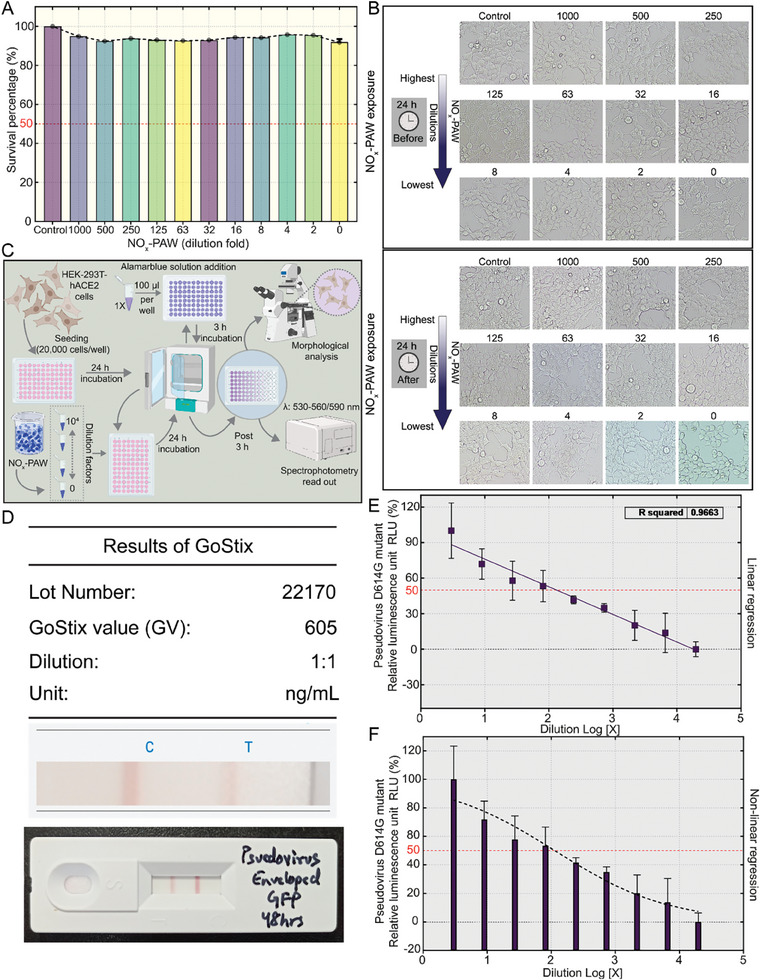
Biocompatibility testing and pseudovirus titration. (A) Alamar Blue assay was used to assess HEK‐293T expressing human angiotensin‐converting enzyme 2 (HEK‐293T‐hACE2) cell viability in various nitric oxide‐plasma activated water (NO_x_‐PAW) dilutions. (B) Microscopy results of the morphological changes in HEK‐293T‐hACE2 cells post‐incubation with NO_x_‐PAW dilutions. (C) Schematic representation of the workflow of the toxicity assessment in hACE2‐overexpressing HEK‐293T cells. (D) Quantification of SARS‐CoV‐2 pseudovirus with the D614G mutation using Lenti‐X GoStix Plus. (E, F) 50% tissue culture infectious dose (TCID_50_/mL) was determined by plotting virus dilution against relative luminescence unit using the Reed–Muench method. Statistical analysis between groups was conducted using ordinary one‐way ANOVA with Dunnett's multiple comparison test. The level of significance was determined, and graphs with non‐significant values were left blank.

To analyze the detrimental effect of NO_x_‐PAW on target cells (HEK‐293T‐hACE2), Alamar Blue assay was conducted. Where the NO_x_‐PAW water was diluted in various dilutions (1000‐, 500‐, 250‐, 125‐, 63‐, 32‐, 16‐, 8‐, 4‐, 2‐, and 0‐fold dilutions) and incubated overnight with HEK‐293T‐hACE2 cells. Post‐incubation morphological changes were observed, and fluorescence was measured to determine cell viability in the different dilutions. The cell survival percentage remained in the range of 92–95% across all NO_x_‐PAW dilutions, and survival was ≈90% in the 0‐fold dilution. However, there was a slight decrease in the fluorescence of the cells treated with the 1000–0‐fold dilutions than that of the control cells, but the difference was not significant (**Figure** **4**A).

Furthermore, HEK‐293T‐hACE2 cells exhibited no notable morphological changes when treated with various dilutions of NO_x_‐PAW, except at the 0‐fold dilution, where slight alterations were observed after 24 h of incubation (Figure 4B). However, cells remained viable (fluorescence data ≈90% cell viability).

Figure 4C illustrates the workflow of the biocompatibility assessment used in this study. Based on the above data, 0‐fold dilution was chosen for subsequent investigations, and it was concluded that the NO_x_‐PAW was not toxic and was biocompatible with HEK‐293T‐hACE2 cells.

### 2.5 Quantification and Titration of the Generated Pseudovirus (SARS‐CoV‐2 D614G Spike, Full Length)

To assess the presence of the pseudovirus (D614G) in the supernatant of HEK‐293T‐hACE2 cells at 48 h post‐transfection, Lenti‐X GoStix Plus was used according to the manufacturer's instructions. Twenty microliters of supernatant containing the pseudovirus were applied to the cassette, and the appearance of the control and test bands was quantified using the GoStix app. The GoStix level was 605 ng mL^−1^ (Figure 4D). Using this formula shown below, the pseudoviral load was calculated as 5 × 10^6^ IFU mL^−1^.

(1) $$\begin{array}{ccc} & & \mathrm{GV}\left(605\right)\times \left(1\times 10^{7}\mathrm{IFU}/\mathrm{ml}\right)/\mathrm{GV}\left(1220\right)\\ & & =\left(5\times 10^{6}\right)\mathrm{IFU}/\mathrm{ml} \end{array}$$

The Glo^TM^ Luciferase Assay was used to determine the 50% tissue culture infectious dose (TCID_50_) of the generated pseudovirus (D614G). The HEK‐293T‐hACE2 cells were exposed to (three‐fold) serially diluted pseudovirus (D614G) containing luciferase gene. After 48 h luciferase substrate was added and readings were recorded. Using the observed relative luminescence unit (RLU) values for each serial dilution, a virus dilution Vs RLU plot was generated and analyzed using linear and non‐linear regression plots to determine TCID_50_, yielding a value of 9.1 × 10^2^ TCID_50_  mL^−1^ (Figure 4E,F).

### 2.6 Inactivation of the Pseudovirus (SARS‐CoV‐2 D614G Spike protein) by NO_x_‐ PAW

**Figure 5:**
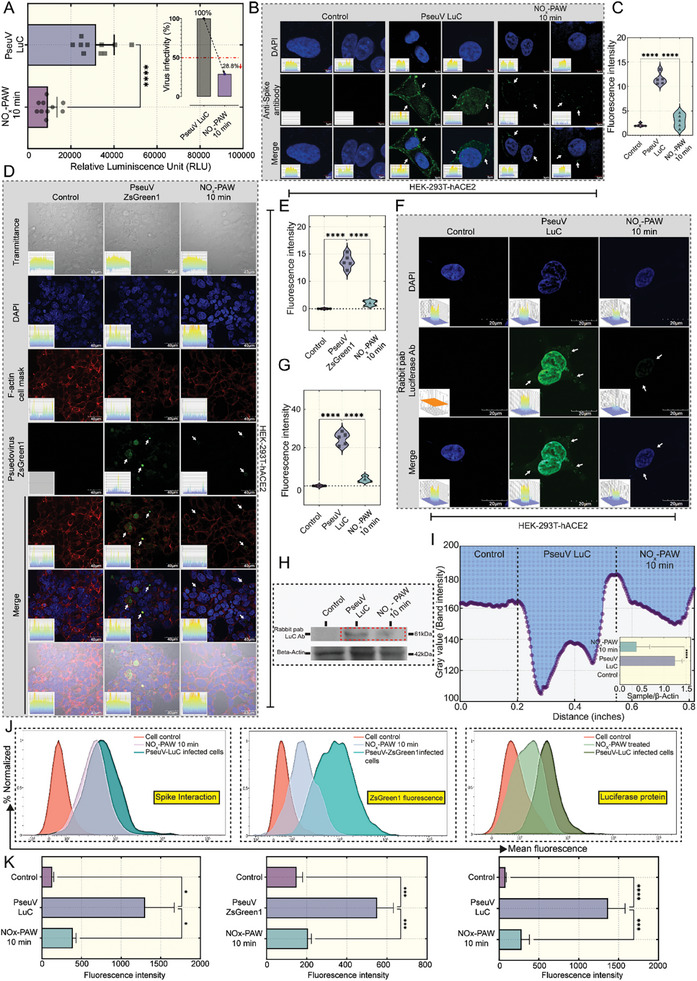
Inactivation of the D614G pseudovirus by nitric oxide (NO_x_)‐plasma‐activated water (PAW) (NO_x_‐PAW). (A) Assessment of the inactivation of the pseudovirus using a luciferase luminescence assay to identify the presence of pseudovirus (D614G, carrying a luciferase reporter gene) infection in HEK‐293T expressing human angiotensin‐converting enzyme 2 (HEK‐293T‐hACE2 cells) (RLU, relative luminescence unit). (B,C) Confocal microscopy analysis and fluorescent intensity analysis of the interaction between the SARS‐CoV‐2 pseudovirus D614G spike protein and HEK‐293T‐hACE2 cells. Groups: Control cells; PseuV LuC, cells infected with pseudovirus D614G carrying a luciferase reporter gene; and NO_x_‐PAW 10 min, cells infected with NO_x_‐PAW treated pseudovirus D614G for 10 min. (D,E) Confocal microscopy and fluorescent intensity analysis of HEK‐293T‐hACE2 cells infected with SARS‐CoV‐2 pseudovirus (D614G, carrying a ZsGreen1 reporter gene).Groups: Control cells; PseuV ZsGreen1, cells infected with pseudovirus D614G carrying a ZsGreen1 reporter gene; and NO_x_‐PAW 10 min, cells infected with NO_x_‐PAW treated pseudovirus D614G for 10 min. Confocal microscopy images of the presence of luciferase in HEK‐293T‐hACE2 cells infected with SARS‐CoV‐2 pseudovirus (D614G, carrying a luciferase reporter gene) and the fluorescence intensities (F,G). (H) Western blot analysis of luciferase in HEK‐293T‐hACE2 cells infected with the pseudovirus (D614G, carrying a luciferase reporter gene) and the Gray value, measured using ImageJ (I). (J) Flow cytometric analysis of the mean fluorescence of spike protein interactions (left), ZsGreen1 (middle), and Luciferase protein (right) with their corresponding graph (K). Statistical analysis between groups was conducted using ordinary one/two‐way ANOVA with Dunnett's or Tukey's multiple comparison tests. The level of significance was determined, and stars (^*^) in the graphs denote *p*‐values (^*^ *p* ≤ 0.05, ^***^ *p* ≤ 0.001, and ^****^ *p* ≤ 0.0001).

Several NO_x_ species were generated by CDBDP in the air phase and then dissolved in water to produce NO_x_‐PAW (Figure 3). We investigated the effect of NO_x_‐PAW on the pseudovirus (D614G) S protein, which serves as a model for the mutated SARS‐CoV‐2 S protein. HEK‐293T‐hACE2 were used as recipient cells to assess the inactivation effects. Cells were divided into two groups: untreated and NO_x_‐PAW treatment for 10 min. Post‐incubation, the average relative luminescence unit (RLU) of untreated and treated HEK‐293T‐hACE2 cells infected with pseudovirus D614G carrying the luciferase gene, were 30241 and 9877, respectively (**Figure** **5**A).

The average RLUs were incorporated into the formula shown below to determine % infectivity, which was calculated to be 29% (Figure 5A).

(2) $$\%\;\mathrm{infectivity}=\frac{9306.6-229}{31640-229}\times 100=29\%$$

Furthermore, the % inhibition was calculated to be 71%.

(3) $$\%\;\mathrm{inhibition}=100-\frac{9306.6-229}{31640-229}\times 100=71\%$$

Immunofluorescence staining was performed to assess the inhibitory effect of NO_x_‐PAW on the pseudovirus (D614G). HEK‐293T‐hACE2 cells were infected with untreated or treated with NO_x_‐PAW for 10 min pseudovirus (D614G) carrying a luciferase reporter gene, alongside a control group. Figure 5B shows the interaction of the pseudovirus (D614G) spike protein with the hACE2 surface receptor in HEK‐293T‐hACE2 cells, resulting in the emission of green fluorescence. However, the fluorescence intensity significantly decreased in the NO_x_‐PAW‐treated group. The fluorescence intensities of these groups were quantified using ImageJ software and are displayed in bar graphs (Figure 5C). Similarly, HEK‐293T‐hACE2 cells were infected with treated NO_x_‐PAW for 10 min or untreated pseudovirus (D614G) carrying the ZsGreen1 reporter gene (100 TCID_50_).

Post‐incubation, confocal microscopy revealed a trend consistent with the above findings of Figure 5B. The NO_x_‐PAW treated group exhibited a significant reduction in the infection rate, as indicated by the decrease in green fluorescence from cells infected with the pseudovirus (D614G) carrying the ZsGreen1 reporter gene compared to that in the untreated group (Figure 5D). This trend was visualized using a bar graph (Figure 5E). Furthermore, the presence of luciferase protein was assessed using western blotting and confocal microscopy to determine the infection rate of the pseudovirus (D614G)‐carrying luciferase in HEK‐293T‐hACE2 cells. Confocal microscopy imaging revealed a significant reduction in luciferase protein levels in HEK‐293T‐hACE2 cells infected with NO_x_‐PAW treated (10 min) pseudovirus (D614G) carrying the luciferase gene compared to those in untreated cells (Figure 5F,G). The western blotting results supported these findings, showing a significant decrease in luciferase protein expression in cells infected with NO_x_‐PAW treated (10 min) pseudovirus group compared to that in the untreated group (Figure 5H; Figure S2, Supporting Information). Figure 5I shows luciferase protein expression intensity in the NO_x_‐PAW treated and untreated groups. Finally, flow cytometry was used to analyze the interaction of the spike and luciferase proteins, as well as ZsGreen1 reporter gene expression in HEK‐293T‐hACE2 cells infected with NO_x_‐PAW treated (10 min) and untreated pseudovirus (D614G) carrying the luciferase or ZsGreen1 reporter gene. Control groups were included for comparison. The findings revealed a significant decrease in the mean fluorescence of the spike protein interaction in the NO_x_‐PAW treated (10 min) group compared to that in the untreated group (Figure 5J,K (left)). Additionally, the mean fluorescence of the ZsGreen1 reporter gene (Figure 5J,K (middle)) and the luciferase protein (Figure 5J,K (right)) was notably decreased in HEK‐293T‐hACE2 cells infected with NO_x_‐PAW treated (10 min) pseudovirus (D614G) carrying the ZsGreen1 or luciferase reporter genes compared to that of untreated cells. The above data suggests that the RNS present in the NO_x_‐PAW generated from CDBD non‐thermal plasma were able to inactivate or neutralize the D614G S‐protein of the pseudovirus and successfully decrease its infection rate.

### 2.7 Effect of NO_x_‐PAW on the Mutated SARS‐CoV‐2 S1‐Domain (D614G)

**Figure 6:**
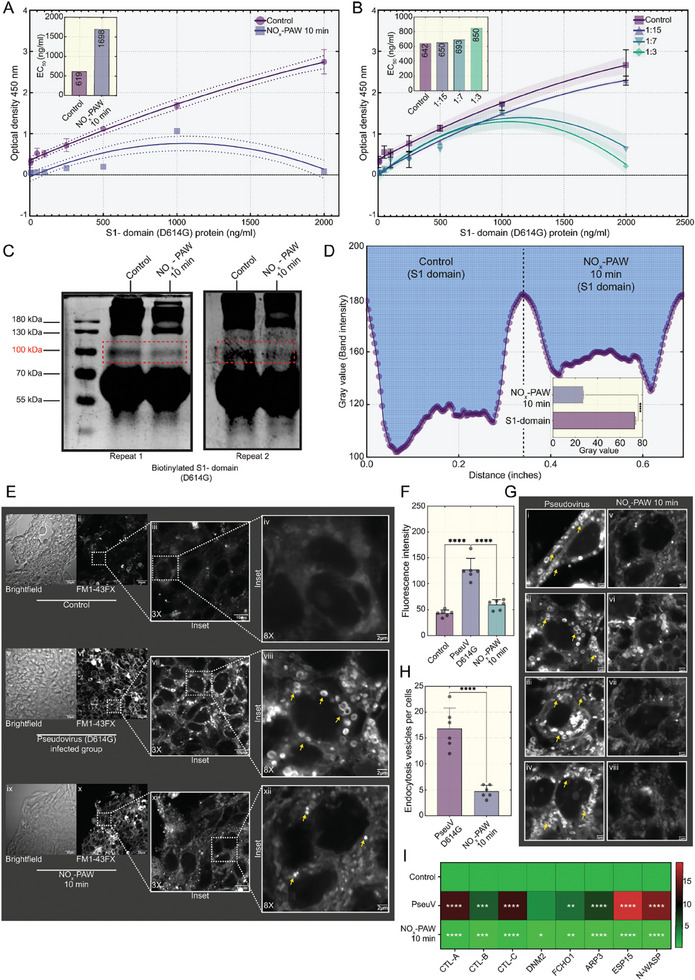
Inactivation of the S1‐domain by nitric oxide‐plasma activated water (NO_x_‐PAW) (A, B) Enzyme‐linked immunoassay of biotinylated S1 protein domain. Groups: control, biotinylated S1‐domain; NO_x_‐PAW 10 min, biotinylated S1‐domain treated with NO_x_‐PAW for 10 min, and treatment of biotinylated S1‐domain with various NOx‐PAW dilutions (1:15, 1:7 and 1:3). EC_50_ values, determined from the graph by plotting across from optical density at 450 nm to various concentrations of biotinylated S1‐domain. (C) SDS‐PAGE analysis of the S1‐domain. Groups: control (the S1‐domain) and NO_x_‐PAW 10 min (S1‐domain treated with NO_x_‐PAW for 10 min). Expression of the S1‐domain in SDS‐PAGE was analyzed using ImageJ (D). (E) Confocal microscopy image of endocytic vesicles in the HEK‐293T‐hACE2 cells, with cell membranes stained with FM1‐43FX dye: (E, i–iv) Control: without pseudovirus, no formation of vesicles; (E, v–viii) pseudovirus (D614G)‐infected cells, formation of vesicles, (E, ix‐xii) NO_x_‐PAW treated 10 min group: pseudovirus (D614G) treated with NO_x_‐PAW for 10 min, reduced formation of vesicles. Endocytic vesicles were measured using ImageJ (F). (G, funding acquisition H) Formation of endocytic vesicles in groups with and without NO_x_‐PAW treatment for 10 min (I) qRT‐PCR analysis of mRNA expression for a gene associated with clathrin‐mediated endocytosis. Statistical analysis between groups was conducted using ordinary‐one/two‐way ANOVA and an unpaired *t*‐test (two‐tailed) with Tukey's multiple comparison tests. The level of significance was determined, and stars shown in the graphs denote *p*‐values (^*^ *p* ≤ 0.05, ^**^ *p* ≤ 0.01, ^***^ *p* ≤ 0.001, and ^****^ *p* ≤ 0.0001). Graphs with non‐significant values were left blank or marked “*ns*.”.

The SARS‐CoV‐2 spike protein is comprised of S1 and S2 domains, with the S1‐domain playing a pivotal role in binding of the hACE2 receptor. The mutation occurring at position 614, where aspartic acid (D) is replaced by glycine (G), situated proximal to the receptor‐binding domain, affects viral infectivity. To elucidate the mechanism of the inactivation of NO_x_‐PAW, the mutated S1‐domain was used as a model for the mutated spike protein. Subsequently, the effect of NO_x_‐PAW on the mutated S1‐domain was investigated to gain insight into its efficacy against mutated viral proteins. Initially, the binding affinity of the S1‐domain (D614G) for hACE2 was assessed using an enzyme‐linked immunosorbent assay (ELISA). Absorbance at 450 nm was recorded for a range of S1‐domain (D614G) concentrations and a dose‐response curve was generated (**Figure** **6**A,B). The half‐maximal effective concentration (EC_50_) of the untreated and treated S1‐domain (D614G) was determined using the plotted curves. The EC_50_ of the untreated S1‐domain (D614G) was 619.3 ng mL^−1^, and the EC_50_ of the NO_x_‐PAW treated S1‐domain (D614G) was 1698 ng mL^−1^. Thus, the EC_50_ of the NO_x_‐PAW treated S1‐domain (D614G) was ≈2.7 times higher than that of the untreated domain (Figure 6A). Furthermore, increasing the volume ratio of NO_x_‐PAW (control, 1:15, 1:7, and 1:3) resulted in increased inactivation, with EC_50_ values of 642.2, 650.1, 693.5, and 850.7 ng mL^−1^ (Figure 6B).

To validate whether NO_x_‐PAW inactivated the pseudovirus (SARS‐CoV‐2 D614G Spike, full length) by damaging the S1‐domain (D614G), the S1‐domain was analyzed for degradation using SDS‐PAGE. After 10 min of treatment, the S1‐domain was significantly more degraded than that in the untreated sample. Typically, the S1‐domain migrated to 100–115 kDa under reducing conditions; the untreated sample displayed a distinct band at 100 kDa, whereas the NO_x_‐PAW treated sample showed a weaker band, indicating potential S1‐domain (D614G) protein degradation (Figure 6C; Figure S3, Supporting Information). Subsequently, ImageJ was used to quantify the protein band intensities, which demonstrated diminished S1‐domain (D614G) expression in the NO_x_‐PAW treated samples (Figure 6D). The above data demonstrated that RNS in NO_x_‐PAW damaged the S1‐domain (D614G), which results in its inactivation effects.

### 2.8 NO_x_‐PAW Inhibits the Interaction of the D614G‐Mutated SARS‐CoV‐2 Pseudovirus and hACE2, Resulting in Reduced Clathrin‐Mediated Endocytosis (CME)

To investigate the effect of NO_x_‐PAW on CME of the SARS‐CoV‐2 variant, the pseudovirus (D614G) was used as a model for a mutated SARS‐CoV‐2 variant carrying the D614G spike mutation. Pseudovirus interaction here mimics viral entry into target cells (HEK‐293T‐hACE2), wherein internalization is facilitated by interactions between the S protein and hACE2, followed by CME. Here, after treating the pseudovirus with NO_x_‐PAW for 10 min, HEK‐293T‐hACE2 cells were infected with treated or untreated pseudovirus (D614G S protein) and incubated for 24 h. Internalization of the pseudovirus into cells was assessed by analyzing the plasma membrane of HEK‐293T‐hACE2 cells using FM 1–43FX membrane staining under a confocal microscope. The results indicated a significant reduction in the internalization (endocytosis) of the pseudovirus treated with NO_x_‐PAW by HEK‐293T‐hACE2 cells compared to that of the untreated pseudovirus (D614G) (Figure 6E). Further analysis of fluorescence intensities corroborated these findings (Figure 6F). Moreover, endocytic vesicles in both the NO_x_‐PAW 10 min treated and untreated cells were quantified using confocal microscopy images, and the vesicle counts were graphically represented. The data indicated a lower incidence of endocytic vesicle formation in the NO_x_‐PAW treated group than in the untreated group (Figure 6G,H). Next, the expression levels of genes linked to CME were assessed using qRT‐PCR (Table S1, Supporting Information). These findings revealed increased mRNA expression of CME‐associated genes in HEK‐293T‐hACE2 cells infected with pseudovirus compared to that in control cells. However, infection with NO_x_‐PAW treated (10 min) pseudovirus resulted in decreased expression of CME‐related genes, similar to that in the control (Figure 6I). These observations indicate that NO_x_‐PAW efficiently inactivated the pseudovirus containing the mutated S protein (D614G), thereby interfering with its binding to hACE2 receptors on HEK‐293T‐hACE2 cells and impeding its internalization into host cells.

### 2.9 Molecular Interaction of RNS in NO_x_‐PAW with the SARS‐CoV‐2 D614G Mutated S1‐Domain: In Silico Approach

**Figure 7:**
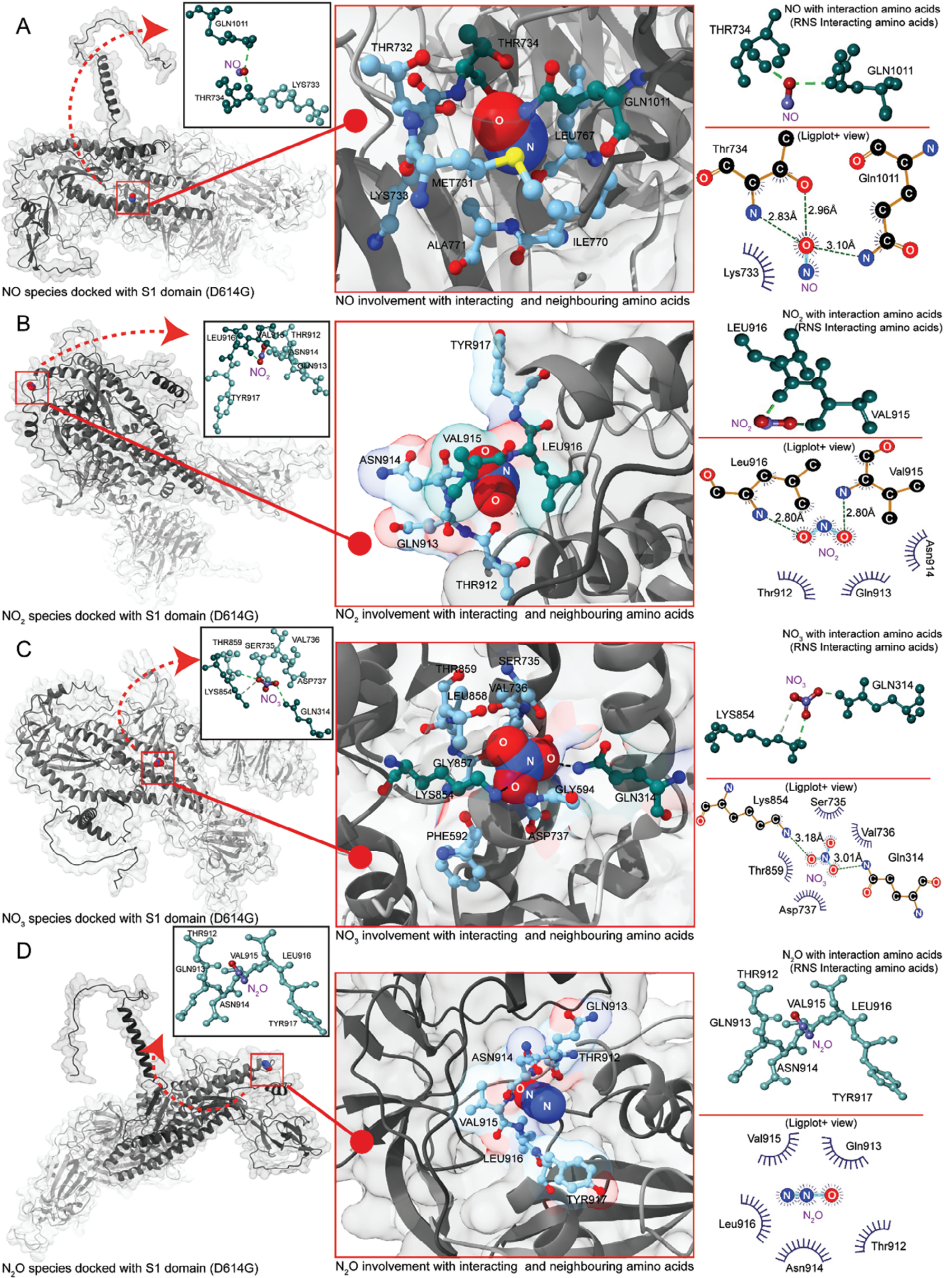
Representation of S1‐domain (D614G) interacting with reactive nitrogen species (RNS), highlighting amino acids that formed covalent bonds. Teal‐colored aa were involved in covalent bonds with RNS. Light blue coloring in the ChimeraX image represents (image in the middle) neighboring and hydrophobic amino acids, while light teal in the DSV image represents hydrophobic residues. The accompanying 2D (ligplot+) (image on the right) interaction image visualizes covalent and hydrophobic bonds, including bond distances. (A) Representation of nitric oxide (NO) species docked with S1‐domain. (B) Depiction of NO_2_ species docked with the S1‐domain. (C) Illustration of NO_3_ species docked with S1‐domain. D) Visualization of N_2_O species docked with the S1‐domain.

Computational analysis was conducted to gain insights into the molecular interactions of RNS (NO_3_, NO, NO_2,_ and N_2_O) with the S1‐domain (D614G). The S1‐domain (D614G) structure was obtained from the best hit of the homology modeling, and its accuracy was verified using a Ramachandran plot (Figure S4, Supporting Information). Molecular docking of RNS and the S1‐domain (D614), obtained from homology modeling (The sequence acquisition and pre‐processing yielded a dataset of 1 048 576 nucleotide sequences of SARS‐CoV‐2 compiled from NCBI, GISAID, COV‐GLUE, and NCBI Virus databases and were curated to remove redundancy and ensure high data quality. A BLAST search against the SARS‐CoV‐2 Spike Glycoprotein reference (UniProt accession: P0DTC2) refined the dataset to include only relevant sequences. The D614G S1‐domain sequence was identified and used for multiple sequence alignment (MSA) using the mmseq2_uniref_env parameter with default settings in a Jupyter notebook. Following MSA and complex prediction with Alphafold2 Multimer v3, the first hit model was selected for further analysis, including a Ramachandran plot generated with UCSF Chimera after loading the.pdb file), which was performed multiple times to ensure accuracy, and the optimal output was used for post‐docking analysis. The most optimal binding site for NO in the S1‐domain (D614G) was determined, which contained: MET731, THR732, LYS733, THR734, LEU767, ILE770, ALA771, and GLN1011 neighboring aa (5 Å radius). A covalent interaction between NO and the S1‐domain (D614) formed because of the sharing of electrons between the oxygen atom of NO with the nitrogen atom (2.83 Å) and oxygen atom (2.96 Å) of THR734 in the S1‐domain. Moreover, the nitrogen atom (3.10 Å) of GLN1011 was involved in a covalent bond with the oxygen atom of NO, whilst LYS733 participated in a hydrophobic interaction (**Figure** **7**A). The binding affinity between NO and the S1‐domain (D614G) was −1.9 kcal mol^−1^ (Figure S5, Supporting Information). Furthermore, the binding affinity of NO_2_ for the S1‐domain (D614) was −3.1 kcal mol^−1^ (Figure S5, Supporting Information). In a 5 Å radius of the NO_2_ binding site_,_ the neighboring residues were THR912, GLN913, ASN914, VAL915, LEU916, and TYR917, among which, nitrogen atoms of LEU916 and VAL915, which had the same distance of 2.80 Å, were covalently bonded to the oxygen atom of NO_2_. Additionally, THR912, GLN913, and ASN914 were involved in hydrophobic interactions (Figure 7B). The best binding sites for NO_3_ were GLN314, PRO592, GLY594, SER735, VAL736, ASP737, LYS854, GLY857, 858LEU, and THR859 were recognized to be the neighboring aa (5 Å). Among them, SER735, VAL736, ASP737, and THR859 were involved in hydrophobic interactions.

Furthermore, the oxygen atoms of NO_3_ formed a covalent bond with the nitrogen atoms of LYS854 and GLN314 (3.18 and 3.01 Å, respectively) (Figure 7C). The binding affinity of NO_3_ with S1‐domain (D614G) was −3.3 kcal mol^−1^ (Figure S5, Supporting Information). Subsequent analysis elucidated that the neighboring aa (5 Å radius) of the N_2_O binding site were involved in hydrophobic interactions between VAL915, GLN913, LEU916, ASN914, and THR912 of the S1‐domain (D614G) and N_2_O (Figure 7D), with a binding affinity of −3.1 kcal mol^−1^ (Figure S5, Supporting Information).

**Figure 8:**
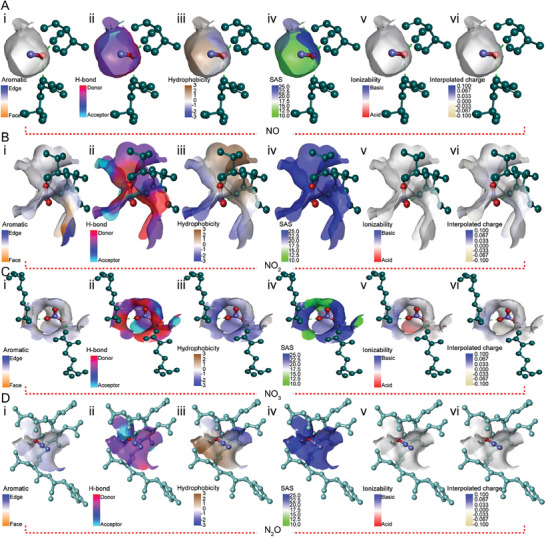
Surface properties of the S1‐domain (D614G) amino acids participating in covalent bonds with reactive nitrogen species The properties displayed include: i) Aromatics, ii) H‐Bonds, iii) Hydrophobicity, iv) SAS (Solvent Accessible Surface), v) Ionizability, and vi) Interpolated charge. (A) The surface of the S1‐domain (D614G) during its interaction with NO. (B) The surface of the S1‐domain (D614G) during its interaction with NO_2_. (C) The surface of the S1‐domain (D614G) upon its interaction with NO_3_. (D) The surface of the S1‐domain (D614G) during its interaction with N_2_O.

Furthermore, the hydrophobic interactions and covalent bonds between the RNS and S1‐domain (D614G) aa residues were analyzed by screening for certain interactions, such as aromaticity, H‐bonding, hydrophobicity, SAS, ionizability, and interpolated charge (**Figure** **8**A–D; Figure S6, Supporting Information).

The strong covalent bonds and hydrophobic interactions between RNS (NO_3_, NO, NO_2,_ and N_2_O) and the amino acids present in the S1‐domain (D614G) may alter the structural integrity and functionality of the S1‐domain (D614G). This could be the reason for the inactivation of the mutated SARS‐CoV‐2 spike protein (S1‐domain), resulting in the decline in the infection rate of the SARS‐CoV‐2 pseudovirus in host cells (HEK‐293T‐hACE2).

## 3 Discussion

Despite a significant decline in the incidence of COVID‐19 worldwide since the peak of the pandemic, the persistent emergence of SARS‐CoV‐2 VOCs is an ongoing threat. Furthermore, these VOCs, which are characterized by mutated forms of the S protein, exhibit enhanced transmission capabilities through infection droplets and contaminated environments (fomites). Additionally, they demonstrate prolonged viability on both hydrophobic and hydrophilic surfaces;^[^ ^9^ ^]^ thus, they exhibit exacerbated transmission dynamics and rates of infection. Among the mutations in SARS‐CoV‐2 that have developed over time, D614G emerged in this study as the predominant non‐synonymous mutation in most VOCs (Figure 1). Effective prevention of transmission necessitates the disinfection of these mutated viral forms within the environment. Therefore, it is imperative to develop efficient strategies for inactivating these variants. In this investigation, using a pseudovirus model, we demonstrated that NO_x_‐PAW was effective in inactivating pseudoviruses harboring the D614G spike protein mutation, thereby inhibiting infection. This observation underscores the potential of NO_x_‐PAW to mitigate the activity of the mutated forms of SARS‐CoV‐2. Viruses require an entry protein to access the host cell.^[^ ^38^ ^]^ The coronavirus spike (S) protein comprises two distinct domains: S1, which is responsible for receptor binding, and S2, which facilitates subsequent membrane fusion events.^[^ ^39^ ^]^ The heightened infectivity of the mutated strain of SARS‐CoV‐2 harboring the D614G mutation within the S1‐domain can be attributed to the disruption of hydrogen‐bond interactions, resulting in the alteration of the conformation of the S protein. This mutation induces a transition of the receptor‐binding domain toward an “open” or “up” conformation, facilitating enhanced binding with the hACE2 receptor. Consequently, this molecular alteration promotes increased infectivity of the virion.^[^ ^40^ ^]^ In this study, the HEK‐293T cell line overexpressing hACE2 was used to mimic host cells. These cells were exposed to pseudoviruses carrying the D614G spike protein that were either untreated or treated with NO_x_‐PAW. Our findings demonstrated that NO_x_‐PAW effectively inactivated the pseudovirus with the D614G spike protein mutation as well as the S1‐domain (D614G), thereby impeding viral entry into the cells.

In the context of both CDBDP‐generated gaseous and PAW, mechanisms involving N_2_O, NO, NO_2_, and NO_3_ are essential for the inactivation of viruses and VOCs. Equation (4) illustrates the production of N_2_O and O by combining N_2_ and O_2_ in the non‐thermal plasma environment generated by the CDBDP. Equation (5) depicts the dissociation of N_2_O into NO and N via electron gain. In addition, NO arises from the dissociation of N_2_ gas within the plasma environment, whereas NO_2_ results from the interaction between NO and O_2_ (Equations (6)–(9)).^[^ ^41^ ^]^ In PAW, interactions between plasma‐generated RNS and water yield reactive species such as NO_x_.^[^ ^35^ ^]^

(4) $$N_{2}+O_{2}\to N_{2}O+O$$

(5) $$N_{2}O+e^{-}\to \mathrm{NO}+N+e^{-}$$

(6) $$N_{2}+O_{2}\to 2\mathrm{NO}$$

(7) $$O_{3}+\mathrm{NO}\to NO_{2}+O_{2}$$

(8) $$O_{3}+NO_{2}\to NO_{3}+O_{2}$$

(9) $$\mathrm{NO}+NO_{3}\to 2NO_{2}$$

Proteins are primary biological targets of radicals owing to their high reaction rate constants.^[^ ^42^ ^]^ Within proteins, tyrosine, and tryptophan exhibit heightened susceptibility to reactions with ONOO^−^, resulting in the formation of 3‐ nitrotyrosine, 6‐ nitrotyrosine, and dityrosine.^[^ ^43^ ^]^ These reactions induce protein aggregation and fragmentation. Similarly, in the present study, the RNS contained in PAW selectively engaged in reactions with amino acid residues within proteins. The in silico approach used in this study elucidated the engagement of RNS in NO_x_‐PAW with the mutated spike protein (S1‐domain). These interactions led to modifications within the S1‐domain, resulting in decreased binding affinity of the S1‐domain (D614G mutation) for hACE2, as well as a reduction in the formation of a protein band at 100 kDa (Figure 6A–D). Thus, the observed decrease in binding affinity for hACE2 and alteration in protein band formation may stem from aggregation or fragmentation of the mutated S1‐domain induced by NO_x_‐PAW treatment, resulting in the appearance of bands of different molecular sizes.

**Figure 9:**
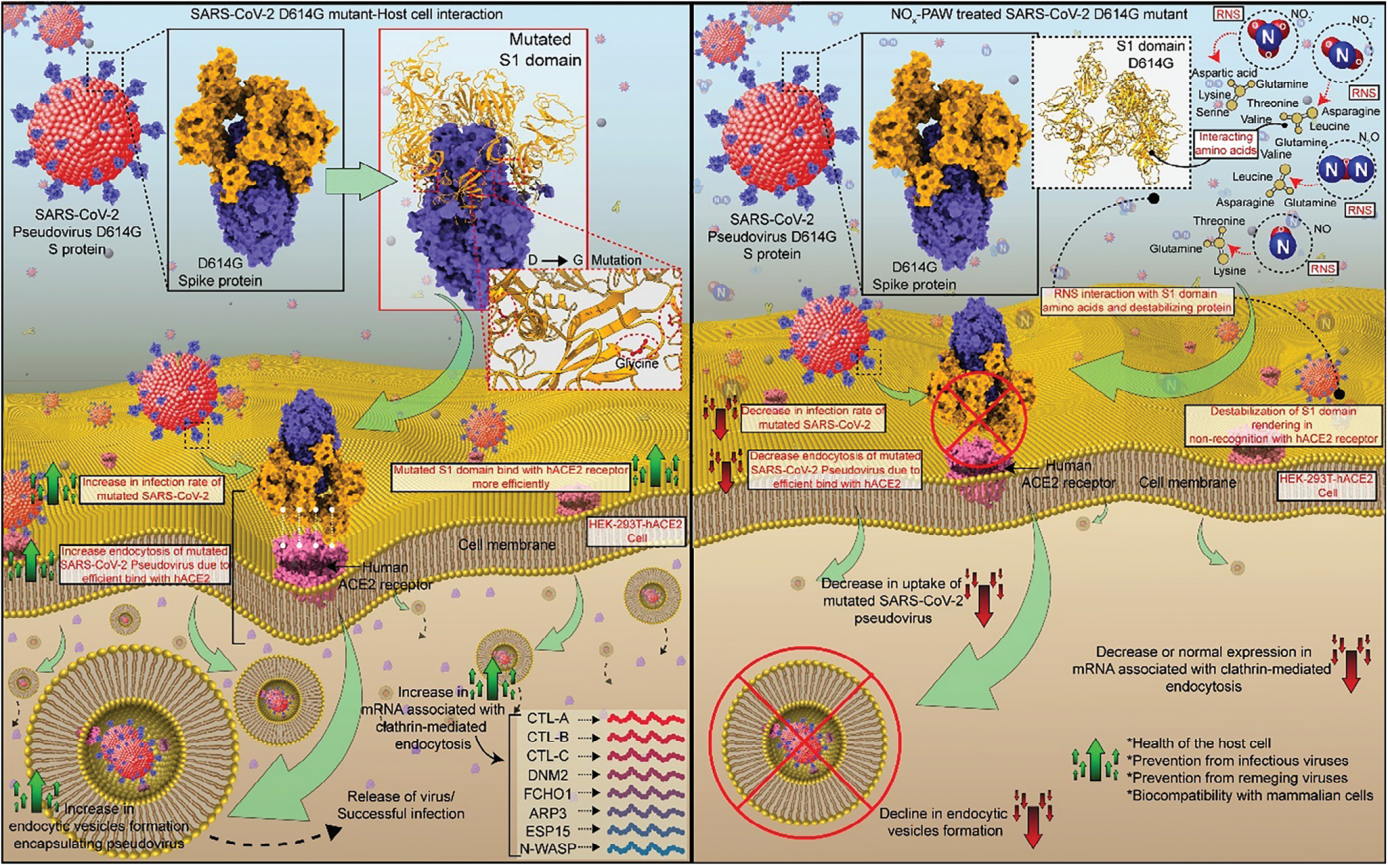
An overview of the mechanism of inactivation by reactive nitrogen species of pseudovirus harboring D614G mutated spike protein, which leads to abrogation of receptor‐mediated endocytosis (clathrin‐mediated endocytosis) by host cells.

Consequently, the reactive species NO, NO_2_^−^, NO_3_^−^, and N_2_O engage with the amino acids in the mutated spike protein (S1‐domain) of the SARS‐CoV‐2 pseudovirus, potentially leading to synergistic effects that enhance the inactivation of the spike protein carrying the D614G mutation (Figure 5). SARS‐CoV‐2 utilizes CME to access host cells, which is a crucial factor in virus infectivity.^[^ ^44^ ^]^ Similarly, in this study, we observed that the D614G SARS‐CoV‐2 pseudovirus employs a CME pathway for cellular entry and subsequent infection.

Furthermore, the bonds formed between the RNS and S1‐domain (D614G) amino acid residues, such as the covalent and hydrophobic bonds between NO and THR734, GLN1011, and LYS733; NO_2_^−^ and LEU916, VAL915, THR912, GLN913, and ASN914; and NO_3_^−^ and LYS854, GLN314, SER735, VAL736, ASP737, and THR859 and the hydrophobic bonds between N_2_O and VAL915, GLN913, LEU916, ASN914, and THR912, resulted in modification of the S1‐domain harboring the D614G mutation, rendering it non‐functional (Figure 7). Therefore, its binding affinity for hACE2 was diminished. Consequently, this reduction in binding affinity for hACE2 correlated with impaired CME and vesicle formation (Figure 6E–I), ultimately resulting in reduced viral infection (**Figure** 9).

The RNS generated in the gas phase, which was subsequently dissolved in DW to yield the final products NO, NO_2_^−^, NO_3_^−^ and N_2_O (Figure 3A–I), are primarily responsible for the inactivation of the S protein (D614G) of the pseudovirus via the S1‐domain (D614G). The NO_x_ species—NO, NO_2_, NO_3_, and N_2_O—each contribute to the inactivation process, earlier literature suggests NO, NO_2_, and NO_3_ play dominant role in pathogen inactivation.^[^ ^30^ ^]^ NO, NO_2_, and NO_3_ exhibit strong binding affinities (such as covalent bonding) and reactivity with critical amino acid in S1‐domain, thereby effectively impairing its binding ability to host receptor leading to viral disruption.^[^ ^45^ ^]^ While N_2_O are present, their roles are less central, with NO showing moderate effectiveness and N_2_O contributing through hydrophobic interactions rather than direct covalent modifications. Compared to reactive oxygen species (ROS), such as hydrogen peroxide (H_2_O_2_) and ozone (O_3_), which are known for their oxidative damage, NO_x_ species demonstrate superior efficacy in this specific context. In this study, the level of ROS did not show significant changes in concentration with varying experimental conditions (Figure 3H). In contrast, observed NO_x_ species (Figure 3), mainly NO, NO_2_, and NO_3_, showed higher effectiveness in inactivating the virus by directly interacting with and modifying viral proteins. This indicates that while oxidative species are potent disinfectants, the NO_x_ species in PAW are particularly effective at disrupting viral proteins and offer a powerful method for pathogen inactivation. This finding implies that RNS can inactivate the mutated form of SARS‐CoV‐2. Similar findings were also observed by Qin et al., in which ONOO^−^ was identified as one of four short‐lived species capable of inactivating GX_P2V and preventing receptor binding of the SARS‐CoV‐2 spike protein.^[^ ^22^ ^]^ The limitation of this study is that the obtained data is mainly based on in‐vitro studies on hACE2‐expressing human cells. Future in‐vivo studies in BSL‐3 settings can be explored to increase confidence in the results.

In comparison to other disinfectants, such as chlorine, ethanol, and bleach, NO_x_‐PAW treatment offers a notable advantage as it can cover a large surface area upon contact (exhibiting a high contact angle) (Figure 3M,N) and does not contaminate the environment with harmful chemicals. As a novel disinfection approach, the storage duration required to retain RNS within NO_x_‐PAW is a crucial factor as it ensures its efficacy in pathogen inactivation. After a storage period of 20 days, NO_x_‐PAW exhibited RNS concentrations >1600 µm (Figure 3F).

## 4 Conclusion

In conclusion, NO_x_‐PAW demonstrated promising capabilities in inactivating SARS‐CoV‐2, with the RNS molecules effectively impeding infection with SARS‐CoV‐2 with mutated spike proteins or VOCs. These RNS molecules inactivate the virus by interacting with the amino acids of the mutated spike proteins and induce modifications. NO_x_‐PAW exhibited storage stability for at least 20 d without residual contamination. Beyond SARS‐CoV‐2, NO_x_‐PAW has the potential for applications in inactivating a wide range of emerging viruses, including both DNA and RNA viruses, as well as their VOCs. Moreover, the practical implication of NO_x_‐PAW, specifically in viral inactivation, offers significant potential in real‐world scenarios such as surface disinfection in hospitals and quarantine zones, viral sterilization of medical equipment, and sanitizing food packages. Additionally, it could be used for wound care as an antimicrobial, in agriculture areas to control plant pathogens and improve crop yields, wastewater treatment as anti‐fouling agent, and degradation of organic pollutants.

## 5 Experimental Section

Detailed methodology is presented in Supporting Information.

### In Silico Analysis and Mutagenesis of SARS‐CoV‐2 Spike Protein

The SARS‐CoV‐2 spike protein structure (PDB ID: 7dzw) was analyzed and mutated using UCSF ChimeraX, with mutations highlighted in red, and then energy was minimized to ensure stability.

### Single Nucleotide Polymorphism (SNP) Analysis

To determine the mutation landscape, genomic variations in spike glycoproteins were identified, annotated, and analyzed using various visualization techniques.

### CDBDP Setup and Electrode Configuration

CDBDP apparatus was used to generate PAW. The setup comprised brass electrodes enclosed within quartz shields, which generated plasma over 90 mm distance. The PAW was produced by exposing deionized water to plasma‐treated air for 30 min under controlled conditions.

### CDBDP Diagnostics

The experimental setup consisted of high‐voltage, current probes, and an oscilloscope to measure plasma energy and power. Spectroscopy was performed using a UV‐NIR spectrometer to identify the reactive species. The Boltzmann plot was used to determine the vibrational and rotational temperatures. An ozone monitor and FTIR system were used to quantify the reactive oxygen and nitrogen species, as well as to calculate the electron density and temperature.

### RONS Determination and Physiochemical Properties of NO_x_‐PAW

The total nitrite in the PAW was determined using a modified Griess approach. H_2_O_2_ concentration was measured using a peroxide detection kit. The functional groups were identified using FTIR spectroscopy. pH, ORP, and conductivity were measured using probes. Contact angles were determined using the sessile drop method.

### Pseudovirus Inactivation Assay

TAKARA provided the SARS‐CoV‐2 S (D614G) pseudovirus carrying a luciferase reporter. HEK‐293T‐hACE2 cells were obtained from ATCC and cultured in complete Dulbecco's Modified Eagle Medium (DMEM). Pseudoviruses were treated with NO_x_‐PAW or left untreated and added to the cells. Cells were assessed for infection after 48 h using the ONE Glo^TM^ Luciferase Assay System. Infectivity and inhibition were measured as percentages (%).

### ELISA

The hACE2 protein was diluted and coated on an ELISA plate overnight. After washing and blocking, the biotinylated SARS‐CoV‐2 S1 protein (D614G) was treated with NO_x_‐PAW or left untreated and added to the coated plate and incubated, followed by the addition of horseradish peroxidase‐conjugated streptavidin. The TMB substrate was added, followed by sulfuric acid, and the absorbance was measured at 450 nm.

### Western Blot Analysis

HEK‐293T‐hACE2 cells were seeded in a 60 mm dish and infected with NO_x_‐PAW treated and untreated pseudovirus (D614G) after 24 h. After 48 h, the cells were lysed, and the protein was quantified using Bradford reagent. SDS‐PAGE was performed, followed by western blotting with a primary luciferase antibody and a secondary HRP‐conjugated anti‐rabbit antibody.

### FM1‐43FX Optical Imaging to Visualize Pseudovirus (D614G) Internalization (Endocytosis)

HEK‐293T‐hACE2 cells were seeded at 10^6^ cells per well on a coverslip. After 24 h, The cells were infected with NO_x_‐PAW treated or untreated pseudovirus (D614G) and incubated for 24 h. After washing, the cells were stained with FM 1–43FX membrane dye, fixed with paraformaldehyde, mounted on a glass slide, and visualized under a confocal microscope.

### Statistical Analysis

Data comparisons between groups were performed using the results of triplicate experiments. Graph plots, such as non‐linear and linear regression plots, and the western blotting results were analyzed using GraphPad Prism 9.3.1 and ImageJ software.

## Conflict of Interest

The authors declare no conflict of interest.

## Author Contributions

P.P. wrote the original draft, developed the methodology, conducted data curation, created visualizations and conceptualization. N.K. contributed to writing, reviewing, methodology, visualization, and editing, as well as conceptualization. T.R.A. wrote the original draft, developed the methodology, and performed data curation. S.S.L. wrote the original draft, developed the methodology, and conducted data curation. S.G. wrote the original draft, developed the methodology, and performed data curation. R.W. contributed to writing, review and editing, as well as funding acquisition. S.K.V. was involved in writing, reviewing, and editing. E.H.C. provided supervision and acquired funding. N.N.K. contributed to writing, review and editing, supervision, funding acquisition, and conceptualization.

## Supporting information



Supporting Information

## Data Availability

The data that support the findings of this study are available from the corresponding author upon reasonable request.
