# Supplementary material for: Inactivation of Pseudovirus Expressing the D614G Spike Protein Mutation using Nitric Oxide‐Plasma Activated Water
Source: Adv Sci (Weinh). 2024 Nov 13;11(48):2411515. doi: 10.1002/advs.202411515 (PMC11672301; doi:10.1002/advs.202411515)
Supplement: Supplementary file 1 — Supporting Information [file ADVS-11-2411515-s001.docx]

**Supplementary file**

**Inactivation of Pseudovirus Expressing the D614G Spike Protein Mutation Using Nitric Oxide-Plasma Activated Water**

**
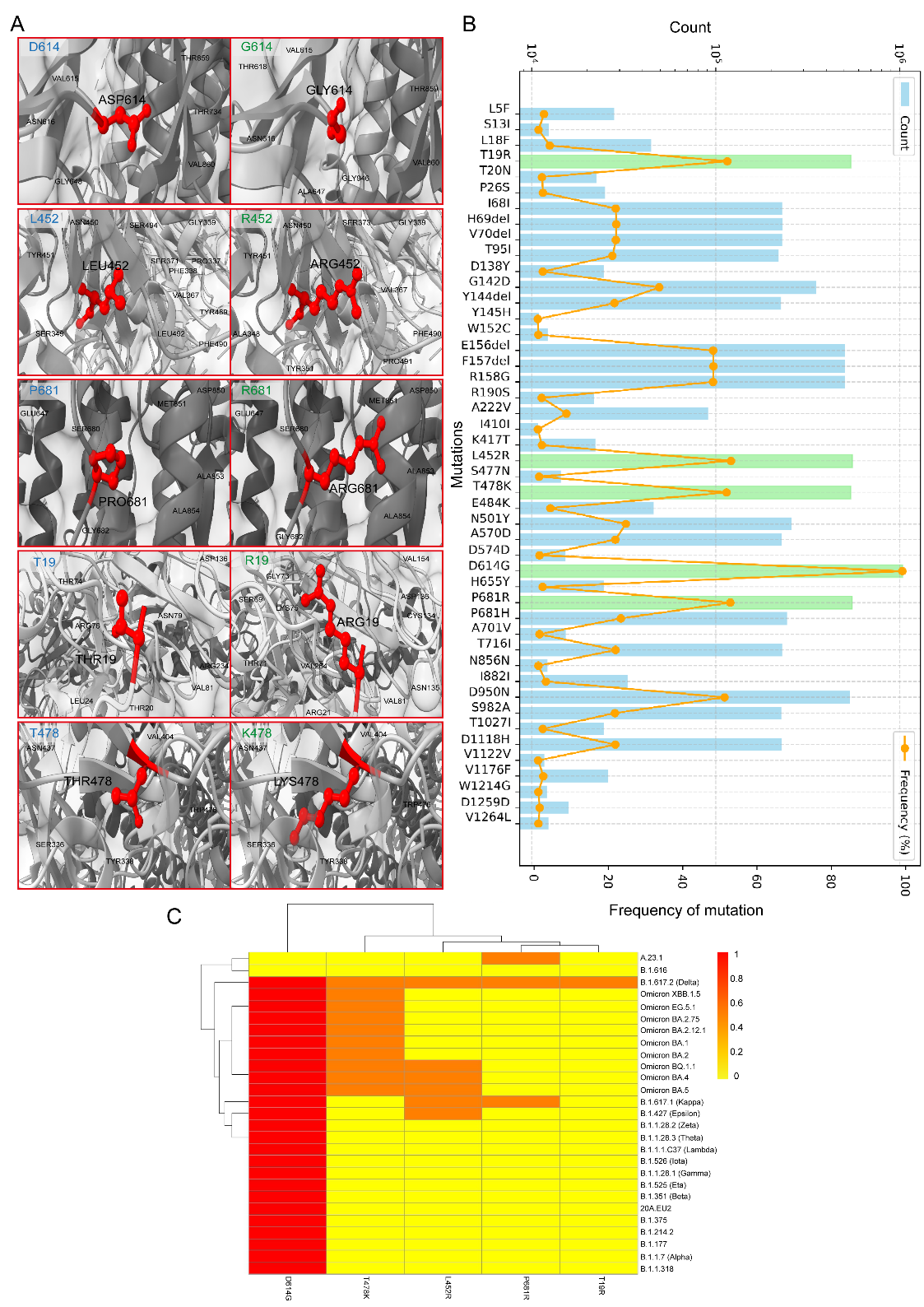
**

**Figure S1. Visualization of Top SARS-CoV-2 Spike Protein Mutations**. (A) Five figures demonstrate molecular structures of the top five mutations identified within the SARS-CoV-2 spike protein. Each subfigure corresponds to a specific amino acid participating in mutation (in red) and highlighting the altered amino acid. The non-mutated structure is labeled in blue (left), whereas the mutated structure is labeled in green (right). (B) The graph shows the five most occurring mutations in the SARS-CoV-2 spike protein. The X- axis marks the amino acid positions affected, while the left Y- axis displays mutation counts, indicating their prevalence. Light green bars highlight the top 5 mutations. The right Y- axis presents mutation frequency as a percentage. (C) A comparative heat map analysis of SARS-CoV-2 spike protein mutations in the top 5 variants is presented. Rows depict amino acid positions along the spike protein sequence, while columns represent the top 5 variants. The color gradient indicates mutation frequency, ranging from low (cool colors) to high (warm colors).

**Figure S2. Western blot analysis of luciferase protein.** (left) Cells exposed to NO_x_-PAW were untreated, and pseudovirus carrying luciferase gene was treated as a reporter. (Right) expression of beta-actin, which was used as a housekeeping gene.

**Figure S3.** Biotinylated S1-domain (D614G) expression under untreated and treated with NO_x_-PAW 10 min.


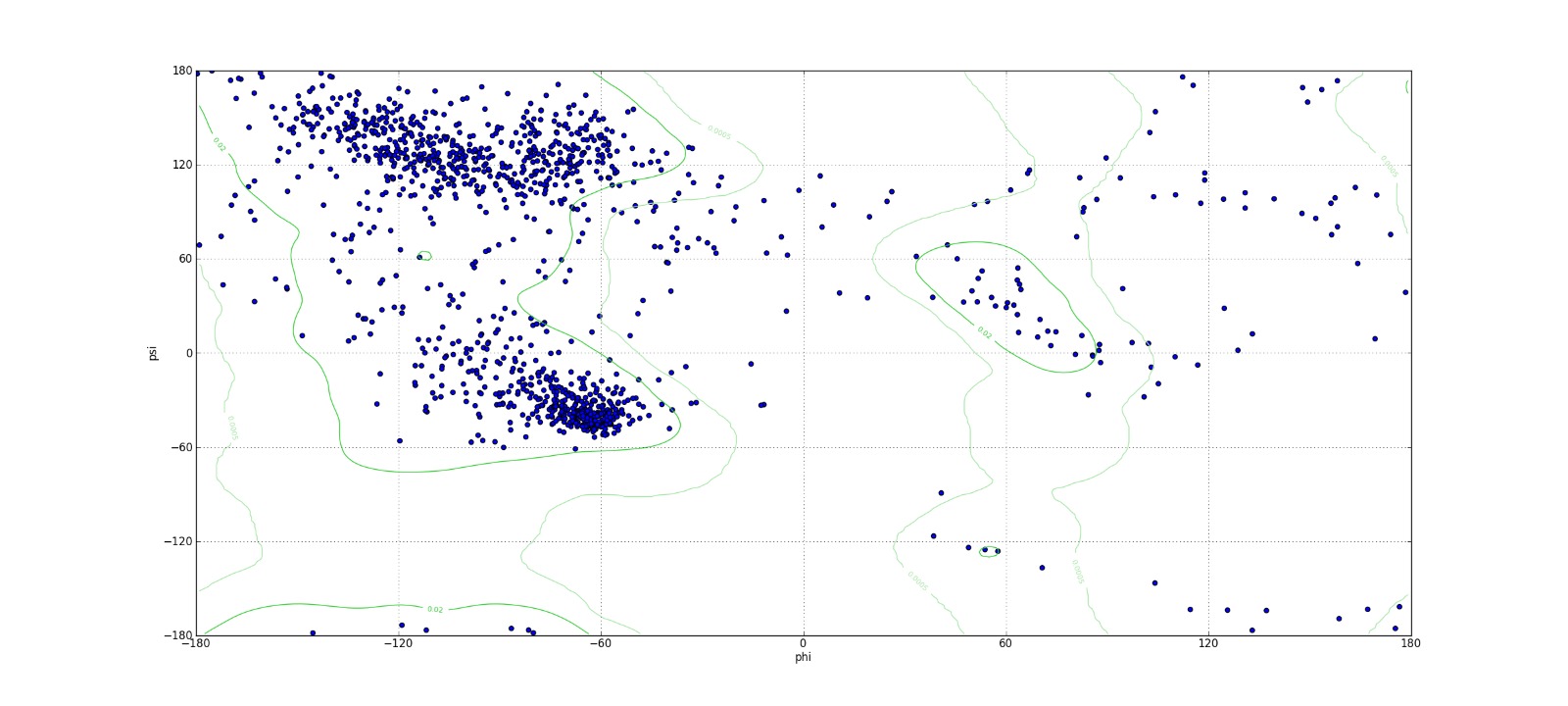


**Figure S4.** Ramachandran plot of S1-domain (D614G).


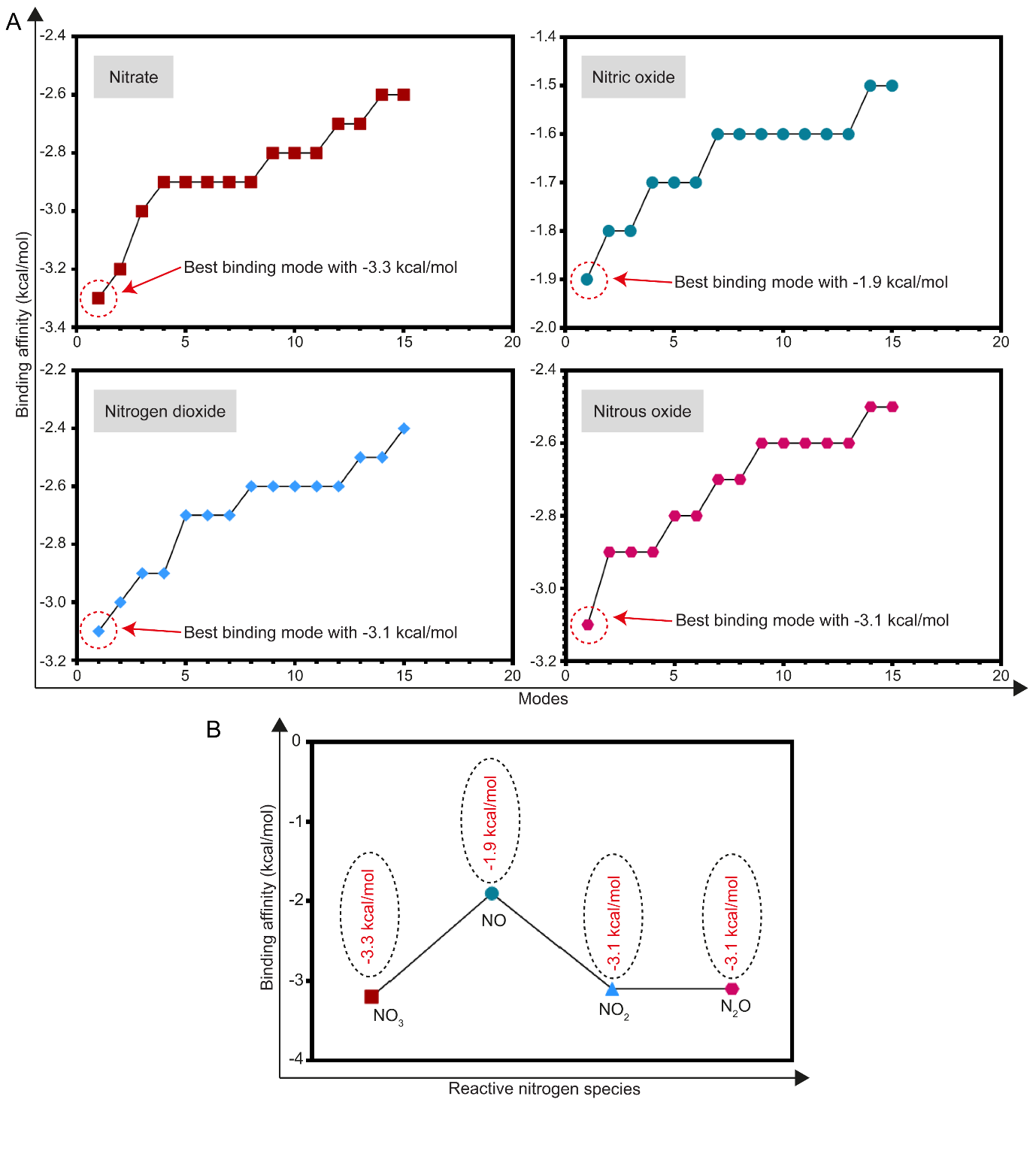


**Figure S5. Binding affinity of reactive nitrogen species with S1-domain** (A) Figure illustrating various binding modes for four reative nitrogen species nitrate, nitrogen oxide, nitrogen dioxide and nitrous oxide plotted against their corresponding binding affinity measured in kcal/mol. On the X- axis different binding modes are displayed, while the Y- axis shows the binding affinity values in kcal/mol. (B) Optimal binding modes for each reactive nitrogen species in relation to their binding affinities.

**
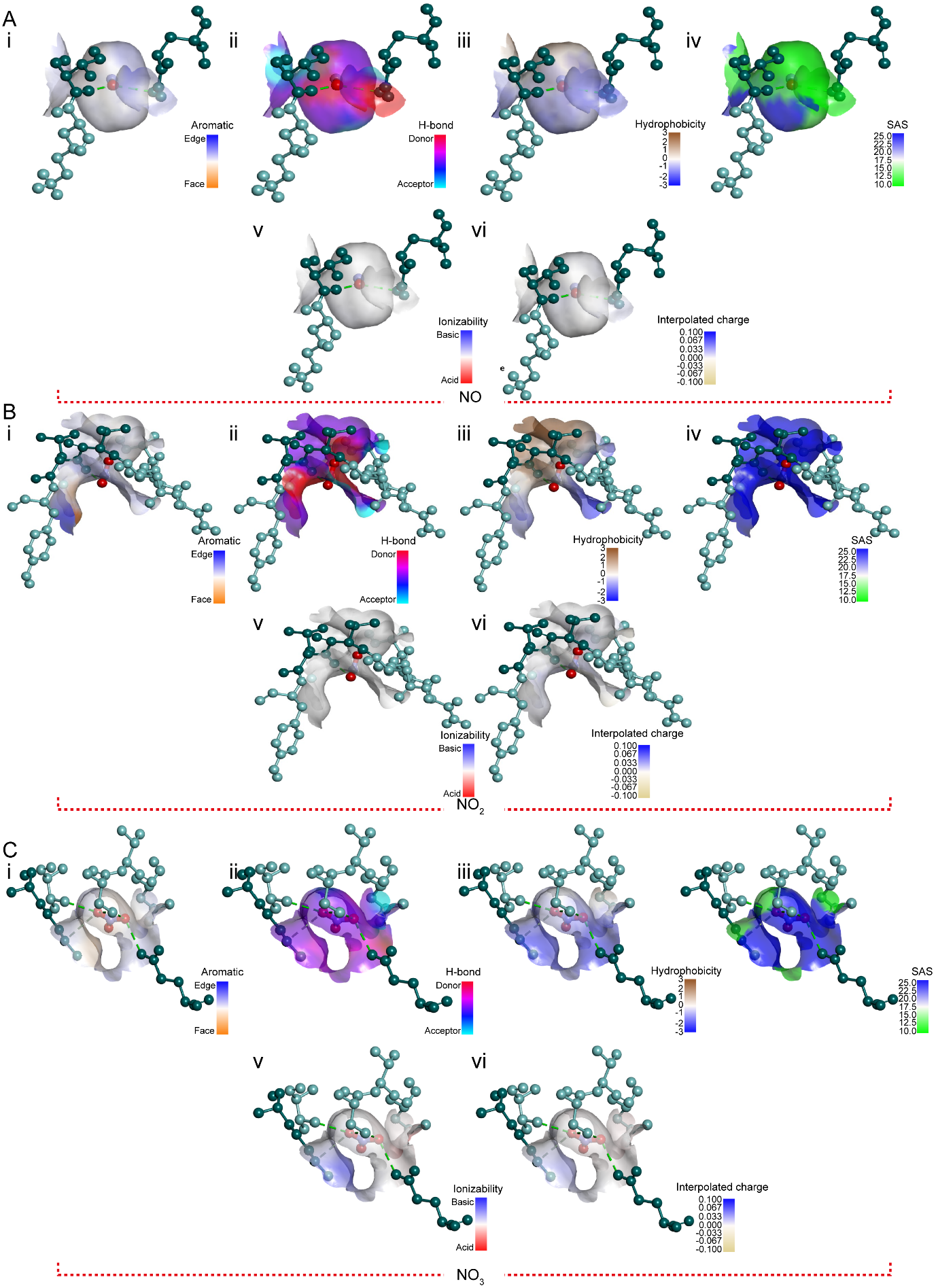
**

**Figure S6. The surface characteristics of amino acids within the S1-domain (D614G) involved covalent bonding and hydrophobic interactions with reactive nitrogen species (RNS).** The properties showcased encompass i) Aromatic, ii) Hydrogen Bonding, iii) Hydrophobicity, iv) Solvent Accessible Surface (SAS), v) Ionizability, and vi) Interpolated Charge. (A) The upper panel demonstrates the S1-domain (D614G) surface interacting with NO. (B) The middle panel shows the surface of the S1-domain (D614G) during its interaction with NO_2_. (C) Lower panel represents the surface of the S1-domain (D614G) upon interaction with NO_3_. D) Illustrates the surface of the S1-domain (D614G) while interacting with N_2_O.

Table S1. Primer sequences involved in the clthrin-mediated endocytosis.

| Gene Name | Sequence (5’-3’) |
| --- | --- |
| Actin-left | GGC ATC CTC ACC CTG AAG TA |
| Actin-right | AGG TGT GGT GCC AGA TTT TC |
| hCLTA-left | TGCCAATTCTCGGAAGCAAG |
| hCLTA-right | ACAAAGGCTTCTTCTGCTGC |
| hCLTB-left | GCGCCAGAGTGAACAAGTAG |
| hCLTB-right | TGCTCTTGGGGTTGAAGTCA |
| hCLTC-left | CAACCAACCAACCACCGATT |
| hCLTC-right | GACGCAACTGACCAACAACT |
| hDNM2-left | AGAGGAGACAGAGCGAATCG |
| hDNM2-right | GGGGATGGCTCTCTTCTTGT |
| hFCHO1-left | ATTTCCTGGAGCCCGATTCA |
| h-FCHO1-right | ATAGAACTTGCGGGGCTCTT |
| hARP3-left | AGCTGCATCTTGGACCTCAA |
| hARP3-right | AGCGCTCCTTTACTGCCTTA |
| hEPS15-left | GGAGGCCCAACTGATCTCTT |
| hESP15-right | AACTGAGCCTTCCCTGACTC |
| hN-WASP-left | TCTGGACGAGATGCACTGTT |
| hN-WASP-right | TGGCTTTGCTCCTTTTCTGC |

**Material and methods**

*Sequence acquisition and pre-processing*

Data comprising 1,048,576 nucleotide sequences of SARS-CoV-2 was compiled from the NCBI virus. These sequences were curated and filtered to exclude redundancy and ensure data integrity. Following this, a BLAST (tblastn) analysis was executed against a reference sequence of the spike glycoprotein of SARS coronavirus 2019 (UniProt accession: P0DTC2.SPIKE_SARS_2). This step refined the dataset, resulting in a final collection of sequences explicitly corresponding to the spike glycoprotein.

*In silico analysis and mutagenesis of SARS-CoV-2 spike protein*

The investigation into the SARS-CoV-2 spike protein structure begins with retrieving SARS-CoV-2 spike protein structures 7dzw from the Protein Data Bank (PDB). Subsequent analysis and mutagenesis of the three-dimensional structure is conducted using molecular visualization software UCSF ChimeraX. The mutation on the spike protein molecular structure is highlighted in red. The spike protein and its mutation were analyzed from all three angles (90 degree turn). Following mutagenesis, energy minimization is performed to ensure the stability of the mutated structure while avoiding steric clashes and structural distortions.

*Single nucleotide polymorphism (SNP) analysis*

Genomic variations within the Spike Glycoprotein sequences were identified using the Genome Analysis Toolkit (GATK). SNPs were detected, and their positions were recorded. The sequences were then translated into protein sequences using EMBOSS Transeq to facilitate mutation verification on the protein level. Mutations were annotated and cataloged along with their respective types, frequencies, and positions. Minor and infrequent mutations (proportion value <0.01) were removed. The annotated dataset, encompassing mutation data of the Spike Glycoprotein, underwent rigorous computational analyses conducted on Google Colaboratory Notebook. Various data visualization techniques were applied to unveil insights into the mutation landscape. A mutation Count and frequency plot was generated, showcasing mutation names on the X- axis and their corresponding frequencies on the right Y- axis and count on the left Y- axis. This facilitated a comprehensive understanding of mutation distribution.

Subsequently, a heat map was constructed, plotting mutation names on the Y- axis against mutation types on the X- axis, each cell color-coded to represent mutation counts. A Sunburst Plot was then generated, portraying mutation types in the inner circle and individual mutations in the outer circle, color-coded based on mutation counts. Additionally, a Variant-wise heatmap was produced, correlating mutation names on the X- axis with variant names on the Y- axis to depict mutation counts per variant. In parallel, an *in-silico* analysis and mutagenesis of SARS-CoV-2 Spike Protein focused on the top 5 mutations. Structural data of the spike protein (7dzw) was extracted from the Protein Data Bank (PDB), analyzed using UCSF ChimeraX, and visually represented, highlighting mutations in red. A Multi-Parameter Bar Graph was plotted, detailing mutation counts and frequencies for the S1-domain as well. Finally, a variant-wise heatmap of the top 5 Mutations was generated, emphasizing the presence of the top 5 mutations across Spike Glycoprotein variants of SARS Coronavirus 2. This heatmap illustrated mutation counts within each variant.

*CDBDP setup and its electrode configuration*

This study employed a Cylindrical Dielectric Barrier Discharge Plasma (CDBDP) apparatus to create plasma treated water (PAW). The setup included four pairs of brass electrodes enclosed within cylindrical quartz sleeves. Each electrode, measuring 1 mm in diameter and 190 mm in length, was alternately connected to either a high-voltage source or ground. The plasma generation zone spanned 90 mm for each electrode pair, with a 1 mm gap between high voltage and ground electrodes. Insulation and encapsulation were achieved using small cylindrical quartz shields and a larger cylindrical quartz cover. PAW was produced by introducing 50 ml of deionized water into the plasma-treated air over a 30 min duration in an open system. All CDBDP treatments were conducted under controlled conditions with a relative humidity of 65 ± 5 % and a room temperature of 27 ± 5° C. The details of the setup of the CDBDP device, including plasma diagnostics, were thoroughly explained in the recently published research paper ^[1]^.

*CDBDP diagnostics*

The voltage across the high-voltage (HV) electrodes was assessed using a Tektronix P6015A HV probe, while the current was simultaneously gauged using the LeCroy CP030 current probe. To capture the current-voltage characteristics, a 434 MHz electronic LeCroy wave surfer Oscilloscope was employed. Utilizing the Eq. (1) and (2), the dissipated energy (E) and the power (P) of the plasma were calculated ^[2]^

E Q×V=$\int_{t1}^{t2} V\left( t \right)I\left( t \right)\mathrm{dt}$(J) (1)

P=F×E=$\frac{1}{t} \times$E(W) (2)

An Ocean Optics HR4000CG-UV-NIR spectrometer connected to a fiber optic with a 400 μm slit diameter was used to conduct a spectroscopic analysis. The main objective was to identify reactive oxygen and nitrogen species (RONS) in the plasma state using optical emission spectroscopy (OES) studies. The existence of RONS was demonstrated by the measured OES intensities, which were expressed in arbitrary units (a.u.). As described in a previously published research work, the vibrational temperature of the CDBDP was ascertained by the use of a Boltzmann plot approach with the N_2_ second positive system (SPS). Together with the generated data, the rotational temperature of the plasma was also calculated using experimental data from the N_2_ SPS peak at 337 nm ^[3]^. Additionally, an examination of the reactive oxygen and nitrogen species (RONS) produced in the gas phase plasma, which includes NO, NO_2_, N_2_O, HNO_3_, and O_3_, was carried out using an ozone monitor (Model 202 Ozone Monitor, B Technologies) and a Fourier-transform infrared spectroscopy (FTIR) system (Bruker Matrix-M5G). OPUS software was used to determine the absolute concentrations of RONS in the gas phase. This provided important information on the electron density and temperature of the CDBDP device. The newly published study presents a thorough examination of the air discharge plasma, offering a thorough comprehension of several physical properties, most notably electron density and temperature ^[1]^.

*Determination of RONS and physicochemical properties of NO_x_-PAW*

Using a modified Griess approach and a commercially available QuantiChrom Nitric Oxide Assay Kit, the concentration of total nitrite (NO₂⁻) in CDBDP PAW was determined. The manufacturer's suggested techniques were followed to determine the NO₂⁻ levels in PAW. In order to assure accuracy, the hydrogen peroxide (H₂O₂) concentration in plasma treated DW was also measured using a QuantiChrom Peroxide detection kit, which relies on the Fe²⁺/Fe³⁺ xylenol orange oxidation process. The manufacturer's instructions were followed. In order to identify functional groups like NO, NO₂, and N₂O, a GX auto-image Fourier transform infrared spectrometer manufactured by PerkinElmer, USA, was used both before and after CDBDP treatment. Additionally, an electrochemical meter set with pH, ORP, and electrical conductivity probes (PHS-3E model from Shanghai INESA Scientific Instrument Co., Ltd.) was used to test the pH, oxidation-reduction potential (ORP), and electrical conductivity of both distilled water (DW) and PAW. Three separate measures were taken following CDBDP treatment. Using the sessile drop method, a 5 μl solution was sprayed onto the surface of glass and polyethylene surfaces to determine the contact angle of DW, bleach, and plasma treated DW.

*Reagents*

NO_x_ and H_2_O_2_ kit (QuantiChrom Nitric Oxide, Peroxide Assay Kit) were obtained from (BioAssay Systems, USA). Cell viability was conducted using Alamarblue assay (Invitrogen, USA). FM^TM^1-43FX membrane stain (Cat # F35355) was sourced from Thermo Fisher Scientific (USA). Antibodies such as human coronavirus S-protein (Cat # 40021-MM07) Sino Biological (USA), Luciferase Antibody (Cat # NB110-17348), Alexa-fluor 488 donkey anti-mouse IgG (Cat # A21202), Alexa-fluor 532 goat anti-rabbit (Cat #A11009), Thermo Fisher Scientific (USA), and APC-conjugated goat anti-rabbit IgG (Cat # SC3846). Recombinant human ACE-2 protein (Cat # ATGP3963) was acquired from NKMAX, South Korea. High binding standard ELISA 96-well plate (Cat # 655061) was acquired from Greiner. Biotinylated S1-Domain (D614G) (Cat # S1N-C82E3) was procured from Acro Biosystems, and HRP conjugated streptavidin (Cat # N100) was procured from Thermo Fisher Scientific (USA). Primers were sourced from DNA Macrogen (Korea). The pseudovirus infections were checked using the ONE-Glo^TM^ Luciferase Assay System (Cat # E6110), which was procured from Promega.

*Generation of pseudotypes lentiviral particles*

Lenti-X^TM^ SARS-CoV-2 Packaging Single Shots (D614G Spike, Full Length) (Cat # 632669) was procured from TAKARA, and pseudovirus containing ZsGreen1 and luciferase as reporter genes was generated using company protocol.

*Cell culture*

HEK-293T-hACE2 (Cat # NR-52511) was acquired from bei resources (ATCC, USA). Cells were cultured in DMEM (Welgene) containing 10 % Fetal Bovine Serum (RD Tech, USA) and 1 % antibiotic (Gibco, Korea).

*Cell viability*

The biocompatibility of HEK-293T-hACE2 cells was assessed using Alamarblue. 20,000 cells/well were seeded in a 96 well plate and incubated for 24 h. Control cells received fresh media, while treatment groups were exposed to various dilutions of NO_x_-PAW in DMEM and incubated. Post 24 h incubation, the treated group was washed with PBS and incubated with alamarblue solution, and absorbance was measured at 530-560/590 nm.

*Quantification of pseudovirus and TCID_50_*

The pseudovirus containing D614G S protein was produced using the company's protocol, and Lenti-X GoStix Plus was used to quantify the pseudovirus. A 20 µl aliquot of supernatant was added to a cassette and incubated for 10 min. Test and control bands appeared, indicating the presence of lentiviral p24. Using the smartphone Lenti-X GoStix plus App, the GoStix value (ng/ml p24) was determined. The actual IFU/ml was calculated from the reference value and the GoStix value of an unknown stock (formula mentioned below) GoStix Value (GV):

Formula: GV (unknown) × (IFU/ml)/GV (reference) = IFU/ml (unknown)

For a 50 % tissue culture infectious dose (TCID_50_), SARS-CoV-2 (D614G) pseudovirus was determined according to the Reed-Muench method. A single-use aliquot from the pseudovirus stock to avoid inconsistency in results due to repeated freezing-thawing cycles. HEK-293T-hACE2 cells were seeded 20,000 cells/well in a 96 well plate. Pseudovirus (D614G) was diluted by serial 3- fold dilutions (9 dilutions in total) with DMEM (- FBS), and a control cell was used as a negative control. Then, the pseudovirus (D614G) was exposed to the target cells in 96 well plates and incubated for 48 h in a 5 % CO_2_ environment at 37º C. Post incubation, the culture supernatants were aspirated gently to leave 100 µl in each well, and 100 µl of luciferase substrate was added to each well and incubated for 2 - 3 min at room temperature. 150 µl of lysate was transferred to a white solid transparent flat bottom 96 well plate for detection of luminescence using a 96 well plate reader (BioTek System). Finally, linear regression and non-linear regression curves were plotted between virus dilutions and relative luminescence unit (RLU).

*Pseudovirus inactivation assay*

Pseudovirus incorporated with SAR-CoV-2 S (D614G) protein and reporter genes luciferase was supplied by TAKARA, and HEK-293T-hACE2 cells overexpressing human angiostatin converting enzyme 2 (ACE2) was procured from ATCC. HEK-293T-hACE2 was cultured in a complete medium containing Dulbecco's modified Eagle's medium (DMEM), 10 % v/v fetal bovine serum (FBS), and 1 % (v/v) penicillin-streptomycin at 37º C in 5 % CO_2_. Pseudovirus (112 TCID_50_) was treated with NO_x_-PAW 10 min, and untreated were mixed with DMEM. Then, the mixture was added to hACE2 overexpressing cells in a 96 well plate and incubated for 48 hours. Post-incubation infections were measured using the ONE-Glo^TM^ Luciferase Assay System. Infectivity and inhibition are described in terms of %, calculated as:

% infectivity = $\frac{(mean [untreated sample] RLUs - mean [blank] RLUs)}{(mean [treated sample] RLUs - mean [blank] RLUs)}$ × 100

% inhibition = 100 – $\frac{(mean [untreated sample] RLUs - mean [blank] RLUs)}{(mean [treated sample] RLUs - mean [blank] RLUs)}$ × 100

*Enzyme-linked immunosorbent assay (ELISA)*

The hACE2 protein was diluted using coating buffer (15 mmol/l Na_2_CO_3_, 35 mmol/l NaHCO_3_, 7.7 mmol/l NaN_3_, pH 9.6) to 0.1 µg/well (1 µg/ml, 100 µl/well) and was coated on high binding standard ELISA 96 well plate. The plate was incubated overnight at 4º C and washed four times with washing buffer (0.05 % TBST, pH 7.4). Then, the wells were blocked using blocking buffer (2 % BSA in washing buffer, pH7.4) followed by incubation for two h at 37º C and washing. Next, 100 µl NO_x_-PAW 10 min and untreated 10 ng/ml - 2000 ng/ml biotinylated SARS-CoV-2 S1 protein (D614G) diluted in sample dilution buffer (0.5 % BSA in washing buffer) and added to each well and incubated for 1 h at 37º C followed by washing. The wells were then incubated with 100 µl of horseradish peroxidase-conjugated with streptavidin for 1 h at 37º C. The plate was washed, and 100 µl of TMB substrate solution was added and incubated at 37º C at dark for 30 min. Post incubation, the reaction was stopped using 50 µl of 1 mol/l sulfuric acid followed by absorbance read out at 450 nm.

*Western blot analysis*

HEK-293T-hACE2 (10^6^ cells/dish) were seeded in a 60 mm dish a day before adding pseudovirus. Post 24 h 112 TCID_50_ pseudovirus (D614G) carrying luciferase gene was treated NO_x_-PAW 10 min and untreated were mixed with DMEM (-FBS) and were added to seeded HEK-293T-hACE2 cells and incubated 48 h. The cells were washed with PBS and collected. Then, the cells were lysed in a lysis buffer (containing protease and phosphatase inhibitors), and the whole cell lysate was collected. Briefly, cells were resuspended in lysis buffer and then lysed on ice with repeated vortexing followed by centrifugation at 13000 rpm, 20 min. The supernatant was discarded, and the pellet was kept at -80º C for further use. Protein estimation was conducted using a Bradford reagent. The proteins were then mixed with 6X loading dye and heated for 10 min at 90º C to perform SDS-PAGE using 5 % (v/v) stacking gel and 10 % (v/v) separating gel. The gel was blotted onto a Polyvinylidene Fluoride (PVDF) membrane and incubated in a blocking buffer of 5 % (w/v) skimmed milk overnight at 4º C. Washing was done between steps using 1X TBST. The PVDF membrane was exposed to primary Luciferase Antibody (Cat # NB110-17348) (1:5000 dilution) diluted in 2.5 % BSA and incubated for 2 h. Then, it was exposed to a secondary antibody, HRP conjugated anti-rabbit antibody (1:10,000 dilution), diluted in 2.5 % BSA, and incubated for 1 h. Then, the substrate was added to the membrane and was observed in the ChemiDoc Imaging System (BioRad).

*SDS-PAGE*

First, the biotinylated S1-domain (D614G) was diluted in sample dilution buffer (1000 ng/ml) and was treated with NO_x_-PAW for 10 min and untreated. Then, they were mixed with 6X loading dye and heated at 90º C for 10 min. The proteins were loaded into a 10 % PAGE gel. In order to intuitively compare the molecular weight change of S1-domain (D614G), a protein marker with a range of 10 - 180 kD was loaded as a reference. The gel bands were finally visualized using Coomassie Blue staining.

*Immunofluorescence staining*

Approximately, 10^6^ cells/ dish HEK-293T-hACE2 cells were seeded in a 60 mm dish the day prior to the experiment. After 24 h, 112 TCID_50_ pseudovirus (1) pseudovirus (D614G) containing luciferase reporter gene and 3) pseudovirus (D614G) containing ZsGreen1 reporter gene) were treated with NO_x_-PAW for 10 min and untreated and mixed with DMEM (-FBS). They were added to the seeded cells and incubated for 48 h. The cells were then rinsed with PBS and fixed in 4 % paraformaldehyde, followed by blocking. Then, the primary antibodies: 1) human coronavirus S-protein antibody 1:200 dilution (for spike protein expression) and 2) Luciferase Antibody 1:600 dilution (for luciferase protein expression) were added to cells infected with pseudovirus (D614G) containing luciferase reporter gene and incubated for 2 h. Thereafter, cells were washed with PBS and labeled with secondary antibodies: 1) Alexa-fluor 488 donkey anti-mouse IgG 1:500 dilution and 2) Alexa-fluor 532 goat anti-rabbit 1:500 dilution and incubated for 30 min, at dark. The cells infected with pseudovirus (D614G) containing the ZsGreen1 reporter gene were self-fluorescent and were stained with an F-actin cell mask 1:1000 dilution and incubated for 30 min followed by washing with PBS. Further, all the treated, untreated, and control groups were stained with 4′,6- diamidino-2-phenylindole (DAPI) to stain the nucleus and observed under Olympus confocal fluorescence microscope.

*FM1-43FX optical imaging to visualize pseudovirus (D614G) internalization (endocytosis)*

HEK-293T-hACE2 cells were seeded in 10^6^ cells/well on a coverslip a day prior to the experiment. 112 TCID_50_ Pseudovirus (D614G) were treated with NO_x_-PAW for 10 min, and untreated, they were infected with the seeded cells and incubated for 24 h. Post incubation, cells were washed with PBS, and FM1-43FX membrane stain (100 µg) was diluted in PBS (without calcium and magnesium) to make a working concentration of 5 ng/ml was added to the cells, followed by incubation for 1 – 2 min. Cells were then washed and fixed with 4 % paraformaldehyde for 10 min. Finally, cells were washed with PBS, and the coverslip was placed on a glass slide to visualize them under a confocal microscope.

*Flow cytometry*

To check the effect of NO_x_-PAW on treated and untreated pseudovirus activity on host cells such as: (1) pseudovirus (D614G) spike interaction and 2) presence of luciferase protein and ZsGreen1 gene in host cells after infected with pseudovirus (D614G) containing luciferase reporter gene and ZsGreen1 reporter gene. Post incubation with NO_x_-PAW treated and untreated pseudovirus with host cells, primary antibodies such as 1) human coronavirus S-protein antibody (for spike protein expression), 2) Luciferase Antibody (for luciferase protein expression) were added to the respective groups, and were analyzed using flow cytometry. Briefly, NO_x_-PAW treated pseudovirus and untreated were mixed with DMEM (-FBS) and added to HEK-293T-hACE2 cells, and incubated for 48 h at 37º C, 5 % CO_2_. The cells were harvested and labeled with desired primary antibodies, followed by tagging with secondary antibodies such as Alexa-fluor 488 donkey anti-mouse IgG and APC-conjugated goat anti-rabbit IgG. Cells infected with pseudovirus (D614G) were directly analyzed, consisting ZsGreen1 self-fluorescent reporter gene. Samples containing antibodies were incubated for 45 min in ice, and the mean fluorescence was analyzed using a BD FACSVerse system.

*Qualitative real-time PCR analysis*

To check the endocytosis of pseudovirus treated with NO_x_-PAW and untreated into the host (HEK-293T-hACE2) cells, the host's cells RNA was extracted after incubating for 24 h followed by RNA extraction using the TRIzol method. Briefly, cells were extracted and resuspended in TRIzol and were processed further according to the company's protocol. The purity of the RNA was measured and was resuspended in 60 µl of 0.1 % diethylpyrocarbonate (DEPC), followed by cDNA preparation. Primer pairs used for qRT-PCR were designed and purchased from DNA Macrogen and are listed (table S1). The effects of the host cell's endocytosis response gene expression towards NO_x_-PAW treated and untreated pseudovirus were analyzed.

*Protein-Ligand Docking: reactive nitrogen species – S1-domain (D614G) molecular interaction*

The molecular interaction of Reactive Nitrogen Species (RNS) with the S1-domain (D614G) of SARS-CoV-2 spike protein was scrutinized via an in-silico approach. For protein-ligand interaction analysis of the S1-domain (D614G), molecular interaction was done individually with NO_3_, NO, NO_2,_ and N_2_O via a molecular docking approach. An interaction study was performed using AutoDock 4.2.6/ AutoDock Tools 1.5.6, with NO_3_, NO, NO_2_, and N_2_O as ligands and the S1-domain (D614G) serving as receptor proteins. The 3D structure of NO_3_, NO, NO_2,_ and N_2_O were generated, and their energy minimization and geometrical optimization were done using the ATB server. Homology modeling was carried out using AlphaFold2 for S1-domain (D614G) structure prediction. The sequence acquisition and pre-processing were carried out for which a comprehensive dataset comprising 1,048,576 nucleotide sequences of SARS Coronavirus 2 was compiled from NCBI, GISAID, COV-GLUE and NCBI Virus databases. These sequences were curated and filtered to exclude redundancy and ensure data integrity. Subsequently, a BLAST search was conducted against a reference sequence of the Spike Glycoprotein of SARS Coronavirus 2019 (UniProt accession: P0DTC2 · SPIKE_SARS2). This step refined the dataset, resulting in a final collection of sequences specifically corresponding to the Spike Glycoprotein. The sequence with the D614G S1-domain was obtained from the blast results. For multiple sequence alignment (MSA), MSA option in notebook was used (the mmseq2_uniref_env parameter was selected and the pairing mode was unpaired paired, which enabled usage of pair sequences from same species + unpaired MSA). For the model type, the auto parameter was utilized, which further called the alphafold2_multimer_v3 for complex prediction. The remaining parameters were all default values. The sequence with the D614G S1-domain was input into the query sequence space, and runtime was activated. After run-time, we got the coverage figure of each sequence, as well as the pdb files, and the images/graphs of the top five hit models of the protein folding. The first hit model was chosen for further experimentation as well as for the Ramachandran plot, UCSF Chimera was used after loading the first hit .pdb file in the program. Python Molecular Viewer (PMV) was used for energy minimization of the S1-domain (D614G). The parameters for RNS were set in Autodock 4.2.6. The grid dimensions for the S1-domain (D614G) were set to 126 x 124x 126, with all the protein receptors having a spacing of 1 Å. The docking was performed for the ligand-receptor complex (NO_3_, NO, NO_2,_ and N_2_O-receptor proteins). Lamarckian genetic algorithms were employed to optimize grid dimensions, while a standard Genetic algorithm was utilized for conducting docking runs. The docking algorithm was configured with a population size of 150 individuals and a predefined limit of 2,500,000 evaluations spanning across maximal generations. Subsequently, post-docking analysis was performed by identifying reactive nitrogen species having the most optimal binding sites characterized by the lowest binding energy and 0 rmsd value. Moreover, ChimeraX, PyMol, LigPlot+, and BIOVIA Discovery Studio Visualizer were employed for conformational clustering and visualization.

**References**

[1] T. R. Acharya, P. Lamichhane, A. Jaiswal, K. Amsalu, Y. J. Hong, N. Kaushik, N. K. Kaushik, E. H. Choi, *Environ. Res.* **2024**, *240*, DOI 10.1016/j.envres.2023.117398.

[2] X. Lu, G. V. Naidis, M. Laroussi, S. Reuter, D. B. Graves, K. Ostrikov, *Phys. Rep.* **2016**, *630*, 1.

[3] P. Lamichhane, T. R. Acharya, J. W. Park, K. A. Amsalu, B. Park, E. H. Choi, *Plasma Process. Polym.* **2023**, *20*, DOI 10.1002/ppap.202300102.
